# CircKPNB1 mediates a positive feedback loop and promotes the malignant phenotypes of GSCs via TNF-α/NF-κB signaling

**DOI:** 10.1038/s41419-022-05149-1

**Published:** 2022-08-09

**Authors:** Yang Jiang, Junshuang Zhao, Yingliang Liu, Juntao Hu, Liang Gao, Hui Wang, Daming Cui

**Affiliations:** 1 Department of Neurosurgery, Shanghai Tenth People’s Hospital, Tongji University School of Medicine, Shanghai, 200072 China; 2 Department of Neurosurgery, Taihe Affiliated Hospital of Hubei University of Medicine, Shiyan, 442000 China

**Keywords:** CNS cancer, CNS cancer

## Abstract

Glioma stem cells (GSCs) are a special kind of cells in GBM showing tumor initiation, self-renewal, and multi-lineage differentiation abilities. Finding novel circRNAs related to GSCs is of great significance for the study of glioma. qPCR, western blotting, and immunohistochemistry were used to detect the expression levels of circKPNB1, SPI1, DGCR8, and TNF-α. The expression of these molecules in GSCs was regulated by lentiviral-based infection. RNA immunoprecipitation assay, RNA pull-down, dual-luciferase reporter, and chromatin immunoprecipitation assays were used to study the direct regulation mechanisms among these molecules. All the MTS, EDU, transwell, neurosphere formation assays, ELDA assays, and xenograft experiments were used to detect the malignant phenotype of GSCs. We found a novel circRNA circKPNB1 was overexpressed in GBM and associated with GBM patients’ poor prognosis. CircKPNB1 overexpression can promote the cell viabilities, proliferation, invasion, neurospheres formation abilities, and stemness of GSCs. Mechanistically, circKPNB1 regulates the protein stability and nuclear translocation of SPI1. SPI1 promotes the malignant phenotype of GSCs via TNF-α mediated NF-κB signaling. SPI1 can also transcriptionally upregulate DGCR8 expression, and the latter can maintain the stability of circKPNB1 and forms a positive feedback loop among DGCR8, circKPNB1 and SPI1. Our study found circKPNB1 was a novel oncogene in GBM and of great significance in the diagnosis and prognosis prediction of GBM and maybe a novel target for molecular targeted therapy.

## Introduction

Glioblastoma (GBM) is the most malignant primary tumor of the central nervous system [1]. Glioma stem cells (GSCs) are a special kind of cells in GBM that have stemness features, can express stem cell markers CD133 and nestin, have the ability to continuous proliferation and multi-line differentiation, and play an essential role in the recurrence and chemoradiotherapy resistance of GBM [2]. In recent years, with the in-depth study of the molecular mechanism of the occurrence and development of malignant tumors, it has been considered that malignant tumors belong to genetic diseases. The overexpression and activation of multiple oncogenes and the loss of function of tumor suppressor genes lead to abnormal differentiation, unlimited proliferation, local invasion, invasion, and distant metastasis of normal cells [3]. Therefore, the search for GBM, especially the oncogenes related to the malignant progression of GSCs, is of great significance for the targeted molecular therapy of malignant tumors.

Circular RNAs (circRNAs) are a class of endogenous non-coding RNAs (ncRNAs) with single-stranded closed-loop structures, which are mainly produced by back-splicing of mRNA precursors [4]. CircRNAs have been confirmed to participate in the occurrence, development, and poor prognosis of malignant tumors by regulating the transcription, translation, variable shear and intracellular distribution of target genes [5]. Our previous study has discovered several circRNAs, such as the overexpression of circARF1, circATP5B and circCHAF1A in GBM and promoting the proliferation and tumorigenesis of GSCs [6–8]. Although there have been increasing studies on circRNAs in GBM in recent years, compared with tens of thousands of circRNAs molecules, the existing research on circRNAs is only the tip of the iceberg, and there are a large number of unknown circRNAs to be further discovered and studied. Therefore, finding new circRNAs related to GBM and GSCs will help improve the role of circRNAs in GBM and provide new molecular targeted therapeutic targets for GBM.

Transcription factor (TF) is one of the critical proteins regulating the malignant phenotype of tumor cells [9]. Spi-1 proto-oncogene (SPI1) is an oncogene that can encode an ETS-domain transcription factor [10]. SPI1 was initially involved in the differentiation and development of myeloid and B lymphocytes [11]. Subsequent studies also reported cancer-promoting effects in malignant tumors such as lung cancer, cervical cancer, and gliomas. For example, a new study found that SPI1 promotes GBM progression by regulating pri-miR-10a processing in an m6A-dependent manner [12]. However, there is no study about whether there is direct action between circRNA and SPI1 in GBM or other tumors.

RNA binding proteins (RBPs) are proteins that can actively bind circRNAs, regulating their back-splicing and synthesis, stability and degradation, intracellular distribution, and so on [13]. Our previous studies found that RBP FMR1 can maintain the stability of circCHAF1A and upregulate its expression in GSCs [8]. DGCR8 was initially reported to mediate the biogenesis of microRNAs (miRNAs) from the primary microRNA transcript [14]. DGCR8 was also reported to decrease lncRNA ZFAT-AS1 expression by attenuating its stability to induce its cleavage and promote the malignant biological behavior of glioma [15]. Besides, it was reported that LINC01198 could maintain the stability of DGCR8 and promotes the proliferation of glioma cells [16]. However, there is no study about the direct regulation between DGCR8 and circRNAs, and it is worth exploring.

In this study, we discovered a novel circRNA hsa_circ_0004796 (also called circKPNB1), the most upregulated circRNAs in GBM compared with the adjacent normal tissues via circNRA sequencing. The Kaplan–Meier survival analysis confirmed that circKPNB1 overexpression was associated with GBM patients’ poor prognosis. Our study aimed to investigate the biological function roles of circKPNB1 in GSCs. We further studied the possible mechanism for circKPNB1 and the direct regulation function between circKPNB1 and SPI1 and DGCR8.

## Results

### CircKPNB1 is upregulated in GBM tissues and correlated with poor prognosis

**Fig. 1:**
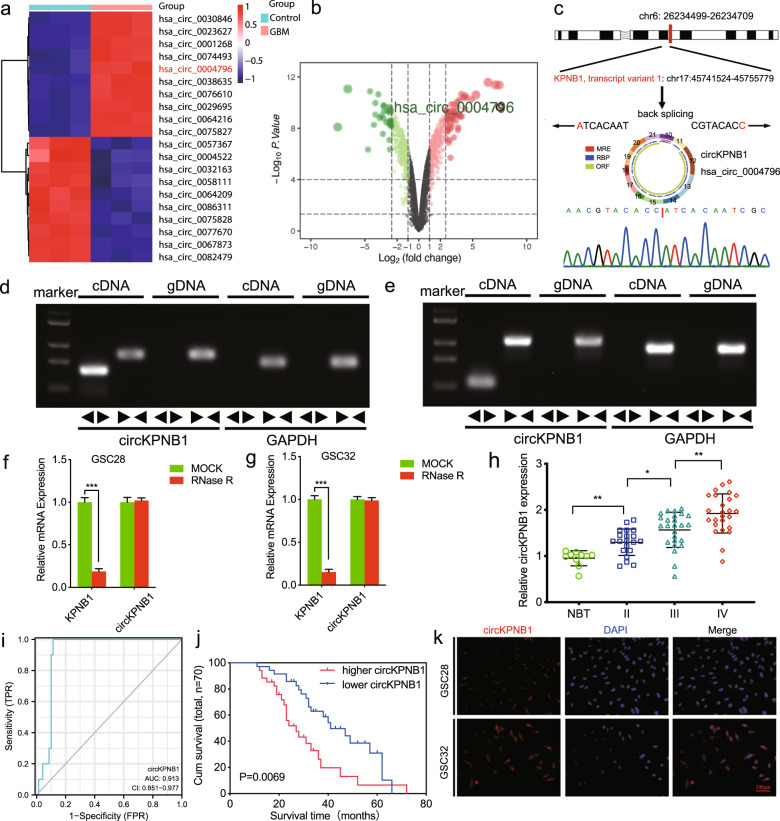
CircKPNB1 is upregulated in GBM tissues and correlated with poor prognosis. **a**, **b** Heatmap (**a**) and Volcano plots **b** showing the differentially expressed circRNAs between three GBM tissues and three adjacent normal brain tissues via circRNA sequencing. **c** The schematic diagram of circKNPB1 and the Sanger sequencing of the back-splicing site of circKPNB1. **d**, **e** The results of agarose gel electrophoresis of qPCR on circKNPB1 expression via divergent and convergent primers. GAPDH was used as a linear control. **f**, **g** The expression of circKNPB1 and KNPB1 after RNase R treatment in GSCs. **h** qPCR showing circKNPB1 expression in WHO grade II, III and IV glioma and normal brain tissues. **i** ROC curves of circKPNB1 in glioma. **j** Kaplan–Meier survival analysis showing the survival rates of circKNPB1 higher or lower expression patients. **k** FISH assays showing the cellular localization of circKNPB1 in GSCs. Scale bar = 200 μm. All data are expressed as the mean ± SD (three independent experiments). *p < 0.05; **p < 0.01; ***p < 0.001.

In order to find the abnormally overexpressed circRNAs in GBM, we performed circRNA sequencing and found hsa_circ_0004796, also named circKPNB1 according to its parental gene, was the highest upregulated circRNAs in GBM (Fig. 1a, b). The schematic diagram showed circKPNB1 was spliced from the KPNB1 gene located at chr17: 45741524- 45755779 and comprised its 10-21th exons (Fig. 1c). The back-splicing site of circKPNB1 was validated by Sanger sequencing (Fig. 1c). To detect whether the head-to-tail splicing of circKPNB1 was caused due to trans-splicing or genomic rearrangements, both the divergent and convergent primers were designed to amplify circKPNB1 in cDNA and gDNA, respectively. The gel electrophoresis results showed that circKPNB1 was detected only in cDNA and indicated that the circular structure of circKPNB1 was produced by reverse splicing (Fig. 1d, e). Besides, the GSCs were treated with RNase R, and the results showed circKPNB1 resisted the digestion of RNase R, while the KPNB1 was digested obviously (Fig. 1f, g). FISH assays showed circKPNB1 was primarily localized in the cytoplasm of GSCs (Fig. 1k). In addition, we also detected circKPNB1 expression in our seventy glioma specimens. The qPCR assays showed that circKPNB1 expressed higher in glioma than in normal brain tissues and expressed even higher in higher WHO grade glioma, with the highest expression in GBM (Fig. 1h). Analysis of ROC curves demonstrated that circKPNB1 may be a potential biomarker of glioma (AUC = 91.3%) (Fig. 1i). The Kaplan–Meier analysis showed that patients with higher circKPNB1 expression have shorter median survival times than those with lower circKPNB1 expression (Fig. 1j). Taken together, circKPNB1 was a novel and upregulated circRNA in GBM, and its expression was associated with the poor prognosis of GBM patients.

### CircKPNB1 regulates the malignant phenotype of GSCs in vitro

**Fig. 2:**
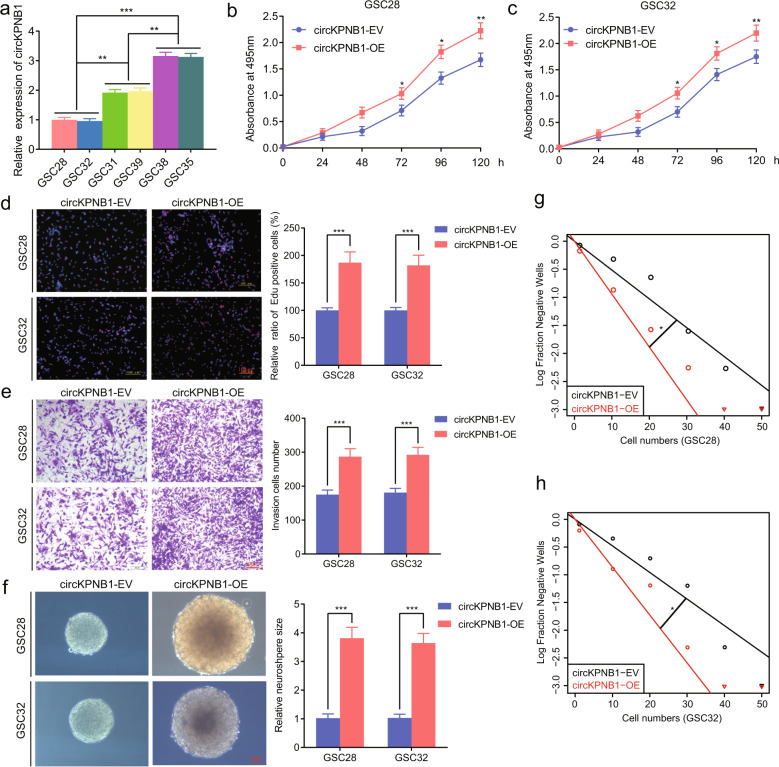
CircKPNB1 overexpression promotes the malignant phenotype of GSCs in vitro. **a** The circKNPB1 expression in six patient-derived GSCs. **b**, **c** MTS assays showing the cell viabilities changes in circKNPB1 overexpressed GSC28 and GSC32. The absorbance value of two groups was compared by two-tailed Student’s *t*-test at different hours, respectively. **d** EDU assays showing the proliferation changes in circKNPB1 overexpressed GSC28 and GSC32. Scale bar = 100 μm. **e** Transwell assays showing the change of invasion cell numbers after circKNPB1 overexpression in GSC28 and GSC32. Scale bar = 50 μm. **f** Neuroshperes formation assays showing the neurospheres formation abilities after circKNPB1 overexpression in GSC28 and GSC32. Scale bar = 50 μm. **g**, **h** Limiting dilution assays showing the self-renewing capacity of GSC4D circKNPB1 overexpressed GSC28 and GSC32. All data are expressed as the mean ± SD (three independent experiments). *p < 0.05; **p < 0.01; ***p < 0.001.

We first successfully cultured six patient-derived primary GSCs from WHO grade IV (GSC28, GSC31, GSC32, GSC35, GSC38, and GSC39). The stem cell markers (CD133, nestin) and multi-lineage differentiation capacities proved that the neurospheres are GSCs (Fig. S1). The expression of circKPNB1 in six patient-derived primary GSCs was detected (Fig. 2a). GSC28 and GSC32 with the lowest expression were used for circKPNB1 overexpression, while GSC38 and GSC35 with the highest expression were used for circKPNB1 knockdown (Fig. 2a). qPCR validated the transfection efficiency of these cells (Fig. S2a, b). All the MTS (Fig. 2b, c), EDU (Fig. 2d), transwell (Fig. 2e), neurosphere formation assays (Fig. 2f) and ELDA assays (Fig. 2g, h) demonstrated the circKPNB1 overexpression obviously promoted the cell viabilities, proliferation, invasion and neurospheres formation abilities of GSC28 and GSC32, while these were all inhibited after circKPNB1 knockdown in GSC38 and GSC35 (Fig. S3). Based on these results, circKPNB1 promotes the malignant phenotype of GSCs in vitro.

### CircKPNB1 can regulate the protein stability and nuclear translocation of SPI1

**Fig. 3:**
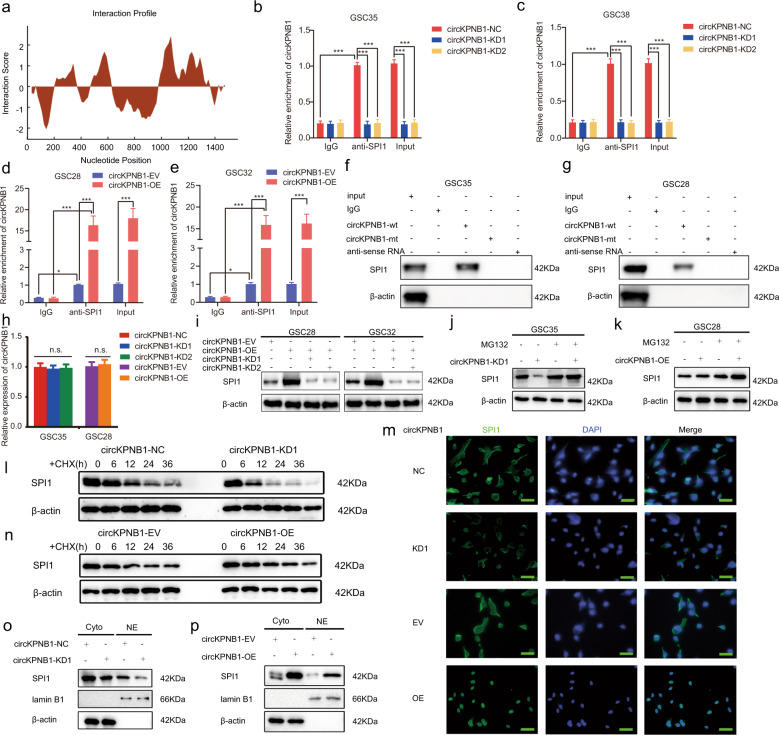
CircKPNB1 can regulate the protein stability and nuclear translocation of SPI1. **a** The binding sites of circKPNB1 on SPI1 protein as predicted by CatRapid. **b**–**e** RIP assays showing anti-SPI1 treatment enriched with circKPNB1 after circKPNB1 knockdown (**b**, **c**) or overexpression (**d**, **e**). **f**, **g** RNA pull-down assays showing the biotinylated circKPNB1 probes pull-down SPI1 protein. **h** qPCR assay showing SPI1 mRNA expression after circKPNB1 overexpression or knockdown. **i** Western blotting showing SPI1 expression after circKPNB1 overexpression or knockdown. **j**, **k** CircKPNB1 silenced (**j**) or overexpressed **k** GSCs treated with or without MG132 (50 µM), and the expression of SPI1 was detected by western blotting. **l**, **n** CircKPNB1 silenced (**l**) or overexpressed **n** GSCs treated with cycloheximide (CHX, 100 ng/ml, and the half-life of SPI1 protein was detected by western blotting. **m** Representative images of immunofluorescence staining of the subcellular distribution of SPI1 in circKPNB1 overexpressed or silenced GSCs. Scale bar = 50 µm. **o**, **p** SPI1 expression in nuclei and cytoplasm of circKPNB1 silenced (**o**) or overexpressed GSCs (**p**) were detected by western blotting. All data are expressed as the mean ± SD (three independent experiments). *p < 0.05; **p < 0.01; ***p < 0.001.

To explore the mechanism of the promoting GBM effect of circKPNB1, we analyzed the possible circKPNB1 binding protein via CatRapid and found SPI1 was the only candidate transcription factor binding circKPNB1 (Fig. 3a). We performed RIP and RNA pull-down assays to study the binding between circKPNB1 and SPI1 protein. RIP assays showed anti-SPI1 treatment enriched with higher circKPNB1 expression than the IgG treatment. Moreover, the anti-SPI1 treatment led to down-regulated enrichment of circKPNB1 in circKPNB1 silenced GSC38 and GSC35 (Fig. 3b, c), while upregulated enrichment of circKPNB1 in circKPNB1 overexpressed GSC28 and GSC32 (Fig. 3d, e). RNA pull-down assays showed biotinylated wild-type circKPNB1 probes could pull down SPI1 proteins while the mutant probe could not (Fig. 3f, g). We furtherly studied the expression of SPI1 after circKPNB1 changes by qPCR and western blotting. qPCR assays showed SPI1 mRNA changed slightly after circKPNB1 overexpression or knockdown (Fig. 3h), while western blotting showed SPI1 was upregulated after circKPNB1 overexpression and reversed after circKPNB1 knockdown (Fig. 3i).

Since circKPNB1 does not affect the mRNA levels of SPI1, we discussed whether circKPNB1 could affect the protein stability of SPI1. The GSCs were treated with a proteasome inhibitor MG-132 after circKPNB1 changes. The results showed that MG-132 treatment obviously recovered SPI1 down-regulation caused by circKPNB1 knockdown and furtherly upregulated SPI1 expression in circKPNB1 overexpressed GSCs (Fig. 3j, k). Besides, CHX assays showed that the half-life of SPI1 protein was prolonged after circKPNB1 overexpression and shortened after circKPNB1 knockdown (Fig. 3l, n). Taken together, our data suggest circKPNB1 can bind SPI1 protein and upregulate its expression via maintaining its protein stability.

We also studied whether circKPNB1 could affect the distribution of SPI1 in GSCs via immunofluorescence. The results showed SPI1 was mainly located in nuclei after circKPNB1 overexpression, while SPI1 was predominantly presented in the cytoplasm after circKPNB1 knockdown (Fig. 3m). Furthermore, western blotting was performed to detect the nuclear and cytosolic distribution of SPI. CircKPNB1 overexpression obviously upregulated SPI1 expression in nuclei and down-regulated its expression in the cytoplasm, while the opposite results were obtained after circKPNB1 knockdown (Fig. 3o, p). Therefore, our data demonstrated that circKPNB1 could promote the nuclear translocation of SPI1.

### SPI1 knockdown can abolish the circKPNB1-induced malignant phenotype of GSCs

**Fig. 4:**
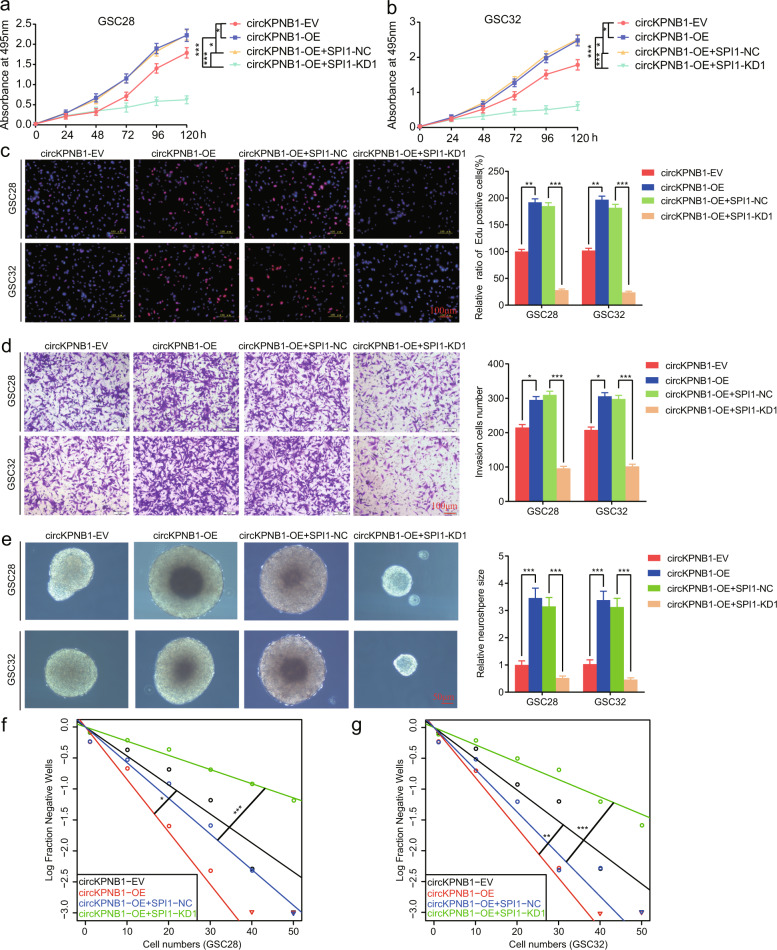
SPI1 knockdown can abolish the circKPNB1-induced malignant phenotype of GSCs. **a**, **b** MTS assays showing the cell viabilities changes in circKNPB1 overexpressed GSCs after SPI1 knockdown. The absorbance value of multiple groups was compared by one-way analysis of variance at different hours, respectively. **c** EDU assays showing the proliferation changes in circKNPB1 overexpressed GSCs after SPI1 knockdown. Scale bar = 100 μm. **d** Transwell assays showing the change of invasion cell numbers in circKNPB1 overexpressed GSCs after SPI1 knockdown. Scale bar = 50 μm. **e** Neuroshperes formation assays showing the neurospheres formation abilities of circKNPB1 overexpressed GSCs after SPI1 knockdown. Scale bar = 50 μm. **f**, **g** Limiting dilution assays showing the self-renewing capacity of circKNPB1 overexpressed GSCs after SPI1 knockdown. All data are expressed as the mean ± SD (three independent experiments). *p < 0.05; **p < 0.01; ***p < 0.001.

In order to demonstrate whether SPI1 was the candidate downstream gene of circKPNB1, the circKPNB1 overexpressed GSCs were furtherly transfected with SPI1 knockdown, and the malignant phenotypes changes were detected by the MTS (Fig. 4a, b), EDU (Fig. 4c), transwell (Fig. 4d), neurosphere formation assays (Fig. 4e) and ELDA assays (Fig. 4f, g). The results showed the cell viabilities, proliferation, invasion, and neurospheres formation abilities of GSC28 and GSC32 were upregulated after circKPNB1 overexpression, while these promoting effects were all inhibited after SPI1 knockdown. Altogether, these results can demonstrate that circKPNB1 promotes the malignant phenotype of GSCs via SPI1.

### SPI1 transcriptionally upregulates TNF-α and activates NF-κB signaling

**Fig. 5:**
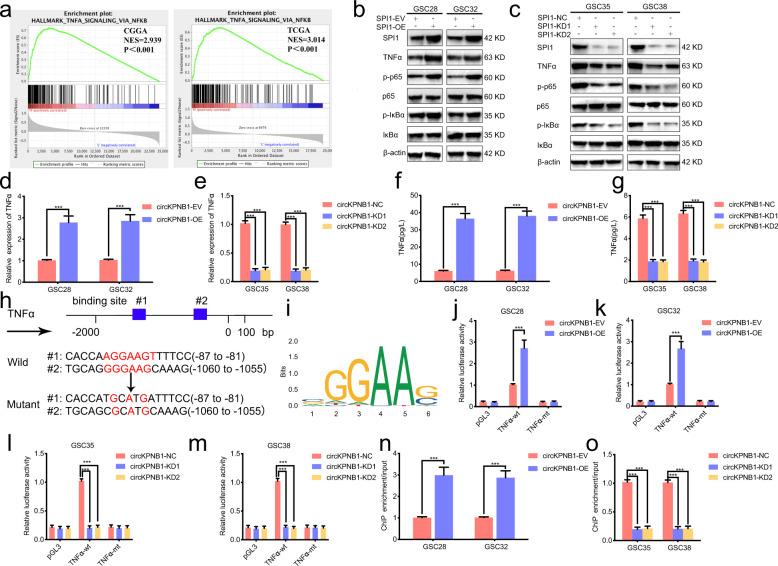
SPI1 transcriptionally upregulates TNFα and activates NF-κb signaling. **a** GSEA analysis showing the enrichment of TNF-α mediated NF-κb signaling in SPI1 higher expression group. **b**, **c** Western blotting showing the expression of TNF-α and the downstream markers of NF-κB signaling after SPI1 changes in GSCs. **d**, **e** qPCR showing the expression of TNF-α mRNA after SPI1 changes in GSCs. **f**, **g** ELISA showing the secretion of TNF-α after SPI1 changes in GSCs. **h** Schematic diagram showing the putative SPI1 binding site in the promoter of TNF-α and sequences of pGL3-TNF-α wild-type and mutant type plasmid. **i** Jaspar database showing the sequence motif of the consensus binding sites of SPI1. **j**–**m** Luciferase reporter assays showing the relative luciferase promoter activities of TNF-α after SPI1 changes in GSCs. **n**, **o** The ChIP qPCR showing the enrichment of TNF-α after anti-SPI1 treatment in SPI1 regulated GSCs. All data are expressed as the mean ± SD (three independent experiments). *p < 0.05; **p < 0.01; ***p < 0.001.

Although SPI1 was reported to promote glioma progression, the exact mechanism was still unclear. We performed GSEA analysis based on TCGA and CGGA datasets and found that TNF-α mediated NF-κB signaling was obviously enrichment in SPI1 higher expression groups (Fig. 5a). Then western blotting (Fig. 5b, c), qPCR (Fig. 5d, e), and ELISA assays (Fig. 5f, g) were all performed to detect the expression and secretion of TNF-α after SPI1 changes. The results showed that the expression and secretion of TNF-α were obviously upregulated after SPI1 overexpression while down-regulated after SPI1 knockdown. Besides, western blotting furtherly detected the downstream of NF-κB signaling, and the results showed that p-P65 and p-IκB were all upregulated after SPI1 overexpression (Fig. 5b) while down-regulated after SPI1 knockdown (Fig. 5c). Moreover, since SPI1 was a transcription factor, we furtherly studied whether SPI1 can transcriptionally upregulate TNF-α expression. The Jaspar database predicted the possible binding sites of SPI1 on the promoter of TNF-α, and the luciferase reporter assays were designed (Fig. 5h, i). The results showed that the relative luciferase activity of pGL3-TNF-α-wt was upregulated after SPI1 overexpression (Fig. 5j, k) and down-regulated after SPI1 knockdown (Fig. 5l, m), while there were no changes in the pGL3-TNF-α-mt group. Besides, ChIP assays also confirmed that anti-SPI1 could lead to enrichment of TNF-α after SPI1 overexpression (Fig. 5n), while the opposite results were obtained after SPI1 knockdown (Fig. 5o). Collectively, these data confirmed that SPI1 transcriptionally upregulates TNF-α expression and activates NF-κB signaling.

### SPI1 can promote the malignant phenotype of GSCs via TNF-α mediated NF-κB signaling

We furtherly study whether TNF-α mediated NF-κb signaling was the possible downstream signaling of SPI1 in GSCs. The neutralizing antibody of TNF-α was designed to block the biological functions of TNF-α. All the MTS (Fig. S4a, b), EDU (Fig. S4c), transwell (Fig. S4d), neurosphere formation assays (Fig. S4e), and ELDA assays (Fig. S4f, g) were performed to detect the SPI1 overexpressed GSCs followed with anti-TNF-α treatment. The results showed that the cell viabilities, proliferation, invasion, and neurospheres formation abilities of GSC28 and GSC32 were upregulated after SPI1 overexpression, while these promoting effects were all inhibited after anti-TNF-α treatment.

### DGCR8 can bind to and maintain the stability of circKPNB1

**Fig. 6:**
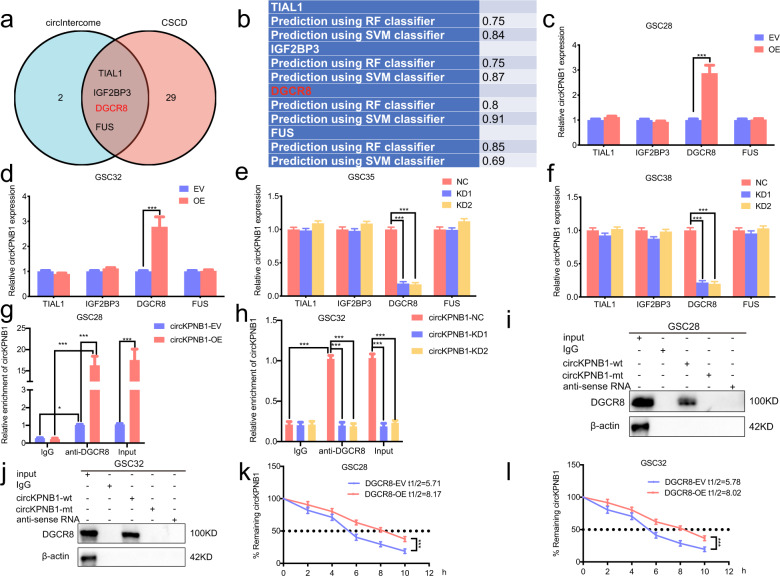
DGCR8 can bind to and maintain the stability of circKPNB1. **a** The RBPs of circKPNB1 were predicted by circIntercome and CSCD. **b** RPISeq showing the binding interaction possibilities between circKPNB1 and four RBPs (TIAL1, IGF2BP3, DGCR8 and FUS). **c**–**f** qPCR showing circKPNB1 expression after overexpression (**c**, **d**) or knockdown (**e**, **f**) of the four RBPs (TIAL1, IGF2BP3, DGCR8 and FUS). **g**, **h** RIP assays showing anti-DGCR8 treatment enriched with circKPNB1 after circKPNB1 overexpression (**g**) or knockdown (**h**). **i**, **j** RNA pull-down assays showing the biotinylated circKPNB1 probes pull-down DGCR8 protein. **k**, **l** RNA stability assays showing the half-life of circKPNB1 in DGCR8 overexpressed GSCs after actinomycin D treatment. All data are expressed as the mean ± SD (three independent experiments). *p < 0.05; **p < 0.01; ***p < 0.001.

Since RBP may participate in the synthesis, stability, degradation, and others of circRNAs, we first predicted the candidate RBP by circIntercome and CSCD and four RBPs (TIAL1, IGF2BP3, DGCR8 and FUS) were found in the intersection (Fig. 6a). Then we analyzed the interaction possibility between circKPNB1 and these four RBPs via RPISeq (Fig. 6b). As shown in Fig. 6b, DGCR8 was the most candidate RBP according to the RF and SVM classifier. Besides, we also performed qPCR assays and found that only DGCR8 can upregulate circKPNB1 expression (Fig. 6c–f). Moreover, we performed RIP assays, and the results showed anti-DGCR8 treatment enriched with circKPNB1 expression. Higher enrichment of circKPNB1 was observed after circKPNB1 overexpression (Fig. 6g), while lower enrichment in circKPNB1 silenced group (Fig. 6h). Moreover, RNA pull-down assays also showed that DGCR8 proteins could be pull-down by the biotinylated wild-type circKPNB1 probes (Fig. 6i, j). In addition, since DGCR8 can upregulate circKPNB1 expression, an RNA stability assay was performed on GSCs using actinomycin D. The results showed that the half-life time of circKPNB1 was obviously prolonged after DGCR8 overexpression (Fig. 6k, l). Therefore, these data confirmed DGCR8 was the RBP of circKPNB1 and upregulated circKPNB1 expression via maintaining its stabilities.

### SPI1 can transcriptionally upregulate DGCR8 and nestin expression and maintain the stemness of GSCs

**Fig. 7:**
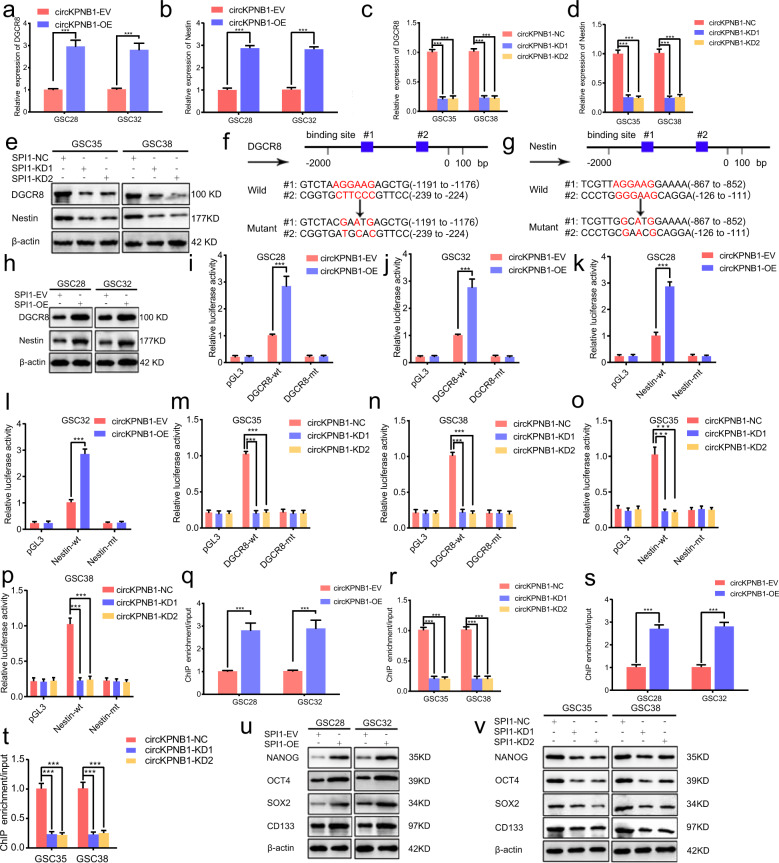
SPI1 can transcriptionally upregulate DGCR8 and nestin expression and maintain the stemness of GSCs. **a**–**d** qPCR showing the mRNA level of DGCR8 (**a**, **c**) and nestin (**b**, **d**) after SPI1 changes in GSCs. **e**, **h** Western blotting showing the expression of DGCR8 and nestin after SPI1 changes in GSCs. **f**, **g** Schematic diagram showing the putative SPI1 binding site in the promoter of DGCR8 (**f**) and nestin (**g**) and sequences of wild-type and mutant-type luciferase plasmid. **i**–**p** Luciferase reporter assays showing the relative luciferase promoter activities of DGCR8 (**i**–**l**) and nestin (**m**–**p**) after SPI1 changes in GSCs. **q**–**t** The ChIP qPCR showing the enrichment of DGCR8 (**q**, **r**) and nestin (**s**, **t**) after anti-SPI1 treatment in SPI1 regulated GSCs. **u**, **v** Western blotting showing the expression of stemness markers (Nanog, OCT4, SOX2, and CD133) of GSCs after SPI1 changes in GSCs. All data are expressed as the mean ± SD (three independent experiments). *p < 0.05; **p < 0.01; ***p < 0.001.

As mentioned above, SPI1 was a transcription factor and we also studied whether SPI1 can transcriptionally upregulate DGCR8 or nestin expression. First, both qPCR and western blotting showing DGCR8 and nestin expression were obviously upregulated after SPI1 overexpression (Fig. 7a, b, h) while down-regulated after SPI1 knockdown (Fig. 7c–e). Then we analyzed the binding sites of SPI1 on the promoter of DGCR8 and nestin on the Jaspar database (Fig. 7f, g). The following luciferase reporter assays showed that the relative luciferase activity of pGL3- DGCR8 -α-wt and pGL3-nestin-α-wt were all upregulated after SPI1 overexpression (Fig. 7i–l), while down-regulated after SPI1 knockdown (Fig. 7m–p). ChIP assays showed that the enrichment of TNF-α and nestin were all upregulated in anti-SPI1 treatment after SPI1 overexpression (Fig. 7q, s) while down-regulated after SPI1 knockdown (Fig. 7r, t). Since nestin was a stem cell marker, we also detected the stemness of GSCs via western blotting. The results showed that SPI1 overexpression obviously upregulates the expression of Nanog, OCT4, SOX2 and CD133 (Fig. 7u), while the opposite results were obtained after SPI1 knockdown (Fig. 7v). These data collectively demonstrated that SPI1 could transcriptionally upregulate DGCR8 expression and form a positive feedback loop among DGCR8, circKPNB1 and SPI1. Besides, SPI1 can transcriptionally upregulate nestin expression and promote the stemness of GSCs.

### DGCR8/circKPNB1/SPI1 feedback loop regulates GBM tumorigenesis in vivo

**Fig. 8:**
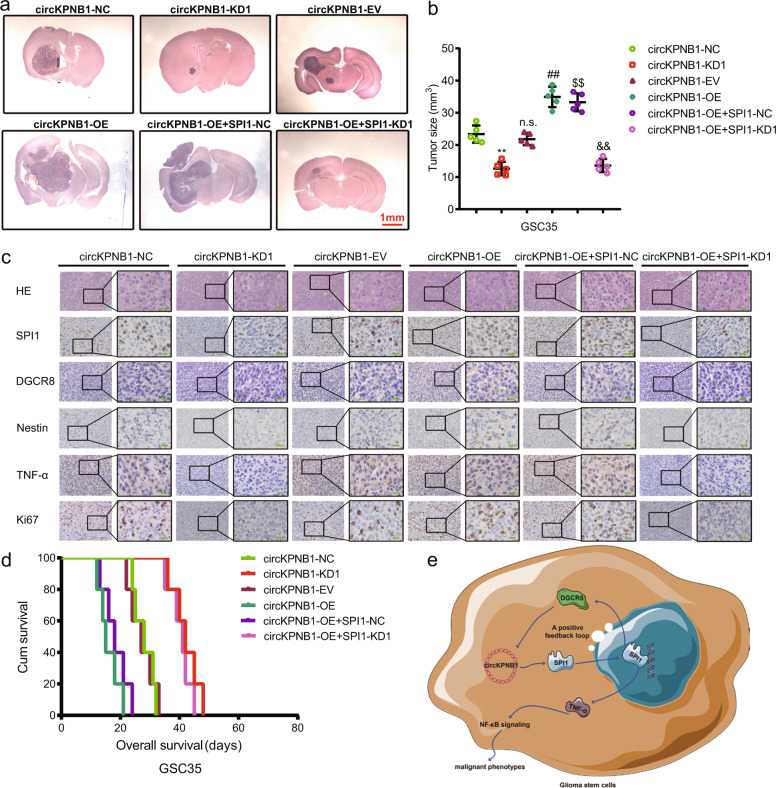
DGCR8/ circKPNB1/ SPI1 feedback loop regulates glioma tumorigenesis in vivo. **a** Representative photographs showing the sizes of intracranial tumors in the coronal position in circKPNB1 or SPI1 overexpressed or knockdown groups. Scale bar = 1 mm. **b** The measured tumor volumes of circKPNB1 or SPI1 overexpressed or knockdown groups. **c** Representative immunohistochemical staining showing the changes in DGCR8, SPI1, TNF-α, nestin, and Ki-67 after circKPNB1 or SPI1 overexpressed or knockdown. Scale bar = 50 μm. **d** Kaplan–Meier survival curves showed the survival times of nude mice in circKPNB1 or SPI1 overexpressed or knockdown groups. **e** Schematic diagram showing DGCR8/ circKPNB1/SPI1 feedback loop regulates glioma tumorigenesis via TNF-mediated NF-κb signaling pathway. All data are expressed as the mean ± SD (three independent experiments). *p < 0.05; **p < 0.01; ***p < 0.001.

We furtherly studied whether DGCR8/circKPNB1/SPI1 feedback loop regulated GBM tumorigenesis in vivo using an orthotopic xenograft model. We found circKPNB1 overexpression resulted in larger tumor volumes than the empty vector, while this promoting effect was reversed after SPI1 knockdown (Fig. 8a, b). However, circKPNB1 knockdown resulted in smaller tumor volumes than the negative control, and this inhibiting effect was also reversed after SPI1 overexpression (Fig. 8a, b). Kaplan–Meier survival analysis showed shorter median survival times after circKPNB1 overexpression while longer median survival times after circKPNB1 knockdown (Fig. 8d). These results were also reversed after SPI1 knockdown or overexpression, respectively. Moreover, the tumor specimens were all stained by immunohistochemistry to show the expression of DGCR8, SPI1, TNF-α, nestin, and Ki-67. The results showed circKPNB1 overexpression led to upregulation of DGCR8, SPI1, TNF-α, nestin, and Ki-67, while they were all down-regulated, followed by SPI1 knockdown. Also, circKPNB1 knockdown resulted in down-regulation of DGCR8, SPI1, TNF-α, nestin, and Ki-67 and they were all reversed after SPI1 overexpression (Fig. 8c). Therefore, as the schematic diagram shows, the positive feedback loop of DGCR8/circKPNB1/ SPI1 led to upregulation of circKPNB1 and SPI1 and promoted the malignant phenotype of GSCs via TNF-mediated NF-κb signaling.

## Discussion

As the most frequent malignant tumor of the central nervous system, GBM’s prognosis is very poor, and the median survival time is even <15 months. The comprehensive treatment effect represented by surgery combined with radiotherapy and chemotherapy is not ideal [13]. Based on the role of oncogenes and tumor suppressor genes, molecular targeted therapy was proposed and has become the most promising way to cure malignant tumors, such as bevacizumab, a targeted inhibitor for VEGF, or trastuzumab, a targeted inhibitor for HER2, etc. [17]. CircRNAs are a special kind of ncRNAs widely distributed and expressed in cells and tissues. Compared with linear RNA, its tissue expression is stable and not easy to degrade [18]. With the wide application of high-throughput sequencing technology, a large number of circRNAs have been found in GBM and other malignant tumors [19]. Our previous study found that circCHAF1A promotes the proliferation and tumorigenesis of GSCs via MDM2-dependent p53 signaling [8]. CircNEIL3 promotes glioma progression and exosome-mediated macrophage immunosuppressive polarization via stabilizing IGF2BP3 [20]. Although circRNAs have become the focus of various malignant tumor research in the recent five years, many unknown circRNAs need to be discovered and studied.

CircKPNB1 is a novel circRNA discovered by circRNA sequencing based on specimens from GBM tissues and the adjacent normal tissues. We performed a qPCR assay to demonstrate its upregulation in glioma and GBM than normal tissues. Moreover, circKPNB1 expression increased further with the increase of WHO grade, with the highest expression in GBM. Besides, we also performed Kaplan–Meier survival analysis and found that higher circKPNB1 expression patients showed obviously shorter median survival time than lower expression patients in GBM. The following functional experiments, such as the MTS, EDU, transwell, and neurospheres formation assays, confirmed circKPNB1 overexpression could promote the proliferation, invasion and neurospheres formation abilities of GSCs, while these promoting effects can be reversed after circKPNB1 knockdown. These results confirm that circKPNB1, as an oncogene, plays a vital role in the tumorigenesis and development of GSCs.

According to published papers, there are three main biological mechanisms of circRNAs. First, since there are many miRNA response elements (MRE) in circRNAs, circRNAs can regulate the expression of downstream target genes via miRNA sponging. For example, circRFX3 contributes to glioma progression through the miR-1179/miR-1229-VASP axis [21]. This is the most widely studied mechanism at present, but it lacks enough innovation. Besides, circRNAs can directly bind RNAs or proteins and regulate their expression, stability and distribution in tumor cells. CircSMARCA5 regulates VEGFA mRNA splicing and angiogenesis in GBM through the binding of SRSF1 [22]. Moreover, although circRNAs belong to ncRNA, many studies have found that circRNAs can encode small molecular peptides, and the laters have the function of regulating malignant tumors. For example, circFBXW7 can encode a 21 kDa small peptide FBXW7-185aa, which can inhibit glioma proliferation [23]. Given the innovation of the research and experimental conditions in this study, we mainly explore the direct regulation between circKPNB1 and downstream target proteins.

In this study, we predicted the downstream target proteins of circKPNB1 via the CatRapid dataset, and the results showed SPI1 was the only candidate transcription factor. The following RIP and RNA pull-down assays demonstrated that circKPNB1 could bind SPI1 protein. Western blotting also demonstrated that circKPNB1 could maintain the stability of SPI1 and upregulate its expression in GSCs without affecting its mRNA expressions. Moreover, the immunofluorescence and western blotting found circKPNB1 promotes the nuclei translocation and possibly affects the transcription of SPI1. SPI1 was reported overexpressed in glioma and could be utilized as a potential diagnostic marker and therapeutic target for glioma [24, 25]. SPI1 could promote GBM progression by regulating pri-miR-10a processing in an m6A-dependent manner [12]. In non-small cell lung cancer, SPI1 exerted an oncogenic role via upregulation of lncRNA SNHG6 and miR-485-3p/VPS45 axis [26]. SPI1 can also be a therapeutic target of cancerous cells, and SPI1 knockdown suppresses aerobic glycolysis and progression of cancer [27]. Therefore, acting as an oncogene, SPI1 is reasonable to be a possible downstream gene of circKPNB1 in promoting the malignant phenotype of GSCs. All the MTS, EDU, transwell and neurospheres formation assays also demonstrated that SPI1 knockdown could abolish circKPNB1-induced malignant phenotype of GSCs.

Although SPI1 is an oncogene in glioma and GBM, the exact cancer-promoting mechanism and signaling of SPI1 are not clear. In our study, we performed GSEA analysis on TCGA and CGGA datasets and found higher SPI1 expression group was enriched with TNF-α mediated NF-κb signaling. This signaling was also one of the critical signalings in tumorigenesis and the development of GBM and other cancers [28]. For example, CLDN4 nuclear translocation induces GBM mesenchymal transition via activating the TNF-α/NF-κB signal pathway [29]. SLC39A7 promotes malignant behaviors in glioma via the TNF-α-mediated NF-κB signaling pathway [30]. Our previous studies also demonstrated that RORA inhibits the proliferation and tumorigenesis of glioma via inhibiting the TNF-α-mediated NF-kB signaling pathway [31].

Therefore, we studied whether SPI1 can promote the malignant of GBM and GSCs via this signaling. All the qPCR, western blotting, ELISA, luciferase report, and ChIP assays demonstrated that SPI1 could transcriptionally upregulate TNF-α expression and secretion and follow activating NF-κB signaling. Moreover, all the functional biological assays demonstrated that anti-TNFα treatment could block the promoting effects of SPI1 overexpression in GSCs. In addition, our study also found SPI1 can transcriptionally upregulate the expression of nestin, which was a GSCs marker and maintain the stemness of GSCs. The results showed that the stemness marker of Nanog, OCT4, SOX2, and CD133 were all upregulated after SPI1 overexpression.

In order to find the reason for circKPNB1 overexpression in GSCs, we searched the candidate RBPs that can bind to circKPNB1 and found DGCR8 can directly bind circKPNB1 via RIA and RNA pull-down assays. DGCR8 acts as an oncogene and participates in the tumorigenesis and progress of several cancers, such as breast cancer [32], ovarian cancer [33] and GBM [12]. Although DGCR8 was initially reported to participate in the processing of miRNAs from pri-miRNA, several studies also found there are regulations between DGCR8 and lncRNAs and circRNAs. As mentioned above, DGCR8 decreased lncRNA ZFAT-AS1 expression by attenuating its stability to induce its cleavage [15]. Circ102049 recruits and distributes DGCR8 protein in the cytoplasm and promotes colorectal liver metastasis [34]. CircPSMC3 inhibits prostate cancer cell proliferation by downregulating DGCR8 [35]. However, there is no study about whether DGCR8 can regulate circRNAs expression. Our study found that DGCR8 overexpression can upregulate circKPNB1 and maintain its stability.

There is no doubt that the occurrence of malignant tumors is not caused by the abnormality of a single gene. The co-abnormal expression of oncogenes and tumor suppressor genes, or a combination of several genes, finally leads to the occurrence and development of malignant tumors. In this study, as a TF, SPI1 can transcriptionally upregulate the expression of DGCR8 and forms a positive feedback loop. This positive feedback loop can lead to the continuous overexpression of DGCR8, circKPNB1, and SPI1 and activation of TNF-α mediated NF-κB signaling in GSCs. Therefore, we believe the positive feedback loop of DGCR8/ circKPNB1/SPI1 promoted the malignant phenotype of GSCs via TNF-α mediated NF-κB signaling. Targeting these molecules may be expected to become an essential target for GBM therapy.

## Conclusion

CircKPNB1 is a novel circRNA that was overexpressed in GBM and correlated with poor prognosis. CircKPNB1 overexpression can promote the proliferation, migration, neurospheres formation abilities and stemness of GSCs. Mechanistically, there is a positive feedback loop among DGCR8, circKPNB1, and SPI1. That is, circKPNB1 can regulate the protein stability and nuclear translocation of SPI1. SPI1 can transcriptionally upregulate DGCR8, and the latter can bind to and maintain the stability of circKPNB1. This positive feedback loop can continuously upregulate TNFα expression and secretion and activates NF-κB signaling in GSCs. Therefore, our study revealed the role of circKPNB1 in GSCs and provided a target for molecular targeted therapy.

## Materials and methods

### Patient specimens and ethical approval

Seventy glioma tissues, including 20 samples of grade II, 25 samples of grade III, and 25 samples of grade IV glioma, were obtained from patients diagnosed with glioma and who underwent surgery in the Department of Neurosurgery of Shanghai Tenth People’s Hospital. Ten normal brain tissue was obtained from patients who suffered from brain trauma. All participants provided written informed consent, and the research was approved by the Ethics Committee of Shanghai Tenth People’s Hospital. The detailed clinical information was listed in Table S1.

### Cell culture

Six patient-derived primary GSCs from WHO grade IV (GSC28, GSC31, GSC32, GSC35, GSC38 and GSC39) were isolated and validated as previously described [8]. The detailed clinical information for these samples is outlined in Table S2. Briefly, freshly resected glioma tissues were dissociated into single cells and maintained in serum-free DMEM/F12 with 2% B27, 20 ng/mL rh-bFGF, and rh-EGF (Gibco, Gaithersburg, MD, USA). The stem cell characteristics of GSCs were validated by detecting the stem cell markers and multi-lineage differentiation capacities. All the GSCs analyzed were cultured with <20 generations and have passed mycoplasma and the short tandem repeat (STR) DNA profiling test.

### CircRNA sequencing

The total RNAs of GSCs were extracted using a Mini-BEST Universal RNA Extraction kit (TaKaRa, Kyoto, Japan). Then the amount and quality of RNAs were detected by Nanodrop (Thermo Fisher Scientific), and the cDNA library was constructed, followed by deep sequencing by the Illumina HiSeqTM2000. CircRNAs with |fold change| ≥ 2 and FDR < 0.05 were recognized as differentially expressed.

### Lentiviral vector construction and transfection

The lentivirus-based overexpression vectors and RNAi-mediated silence of circKPNB1, SPI1, and DGCR8 were all constructed by Gene-Chem (Shanghai, China). After selecting puromycin (Sigma, Santa Clara, CA, USA) at a concentration of 10 μg/ml for 15 days, qPCR and western blotting were used to validate the lentivirus transfection and efficacy [8]. The sequences of all siRNAs are listed in Table S3.

### qRT-PCR (real-time quantitative reverse transcription PCR)

Real-time PCR was performed as previously described [8]. Briefly, the total RNA of GSCs and tissues was extracted as mentioned above. Then Prime-Script RT Master Mix (TaKaRa) was used to synthesize the first-strand cDNA. Finally, the SYBR Green Master Mix (TaKaRa) was performed under PCR LightCycler480 (Roche Diagnostics, Basel, Switzerland). The β-actin was used as an endogenous control. Primers used in this study are listed in Table S4.

### RNase R assay

RNase R was used to eliminate the effect of linear RNAs and confirm the circular structures of circRNAs. Briefly, 10 μg RNA total RNA was incubated with 40U RNase R (Epicentre Technologies, Madison, WI, USA) for 30 min at 37 °C. Then qPCR was performed to detect the expression of linear RNAs and circRNAs.

### Western blotting

Western blotting was performed as previously described [8]. First, the total cell protein extraction kit (KeyGen Biotechnology, Nanjing, China) was used to isolate the total protein of GSCs. The nuclear and cytoplasmic protein was isolated using a NE-PER™ Nuclear and Cytoplasmic Extraction Reagents (Thermo Fisher Scientific). Then, the proteins were separated by 4 to 20% SDS-PAGE (Genscript, Nanjing, China), transferred onto polyvinylidene difluoride (PVDF) membranes, and blocked with 2% bovine serum albumin (BSA, KeyGen Biotechnology) and incubated with the primary antibodies at 4 °C overnight, followed with secondary antibodies (ProteinTech, Chicago, Illinois, USA) incubation. The chemiluminescence ECL kit (Beyotime Biotechnology, Beijing, China) visualized the bands. All results were quantified by ImageJ software (National Institutes of Health, Bethesda, MD, USA).

### MTS assay

MTS assay was performed using the CellTiter 96® AQueous Non-Radioactive cell proliferation assay kit (Promega, Madison, WI, USA) according to the manufacturer’s instructions. Briefly, GSCs were cultured in 96-well plates at a 1 × 10^3^ cells/well density for 24, 48, 72, 96, or 120 h. After incubation, 20 μl MTS was added to each well for 3 h at 37 °C. An ultraviolet spectrophotometer (ThermoFisher Scientific, Waltham, MA, USA) was used to detect the absorbance at 495 nm.

### EDU assay

According to the manufacturer’s instructions, the proliferation of GSCs was detected using an EDU assay kit (Beyotime, Biotechnology). Briefly, the GSCs were seeded into 24-well plates at 1 × 10^5^ cells/well for 20 h. 10 µM EDU reagent was added and incubated at 37 °C for 2 h. Finally, a laser scanning confocal microscope (Olympus) was used to photograph the images, and the percentage of EDU-positive cells was calculated.

### Transwell assay

The transwell assay was performed as previously described [36]. Briefly, 3 × 10^4^ GSCs were seeded into the upper chamber (Corning, Corning, NY, USA) and pretreated with a Matrigel filter (BD Biosciences, San Jose, CA, USA). The lower chamber was treated with a 20% fetal bovine serum medium. After 20 hours of incubation, 4% paraformaldehyde was used to fix the invaded cells. Finally, the cells were stained with crystal violet (Beyotime, Biotechnology), photographed and counted using a light microscope (Olympus).

### Neurosphere formation assay

The neurosphere formation assay was performed as previously described [8]. Briefly, 200 GSCs were seeded in 24-well plates for 7 days. The formed neurospheres were photographed, and the relative neurosphere sizes were calculated under a light microscope (Olympus).

### In vitro limiting dilution assay

As previously described, the in vitro limiting dilution assay was performed [8]. The GSCs were seeded in 96-well plates at a density of 1, 10, 20, 30, 40, or 50 cells/well, with 10 replicates for each density. The neurospheres number was counted after 7 days. The neurosphere synthesis efficiency was calculated by the Extreme Limiting Dilution Analysis (http://bioinf.wehi. edu.au/software/elda) [37].

### RNA immunoprecipitation (RIP) assay

The RIP assay was performed via the EZ-Magna RIP RNA-binding Protein Immunoprecipitation Kit (Millipore, Darmstadt, Germany) as previously described [8]. Briefly, GSCs were lysed in RIP buffer, incubated with magnetic beads conjugated with anti-SPI1, anti-DGCR8 antibodies or negative control IgG (Abcam). Then the protein-RNAs complex was immunoprecipitated, and RNAs were isolated with proteinase K. Finally, RNAs were purified, and qPCR was used to check the circKPNB1 expression in the precipitants.

### RNA pull-down assay

According to the manufacturer’s suggestions, the RNA pull-down assay was performed via the Pierce Magnetic RNA Protein pull-down Kit (Thermo Fisher Scientific). Briefly, the biotinylated wild or mutant type circKPNB1 probes, positive control (input), and negative control (antisense RNA) were used to label RNA and pull down the RNA-protein complex. Then the complex was added with magnetic beads, and the proteins were immunoprecipitated. Finally, the proteins were purified, washed, boiled and detected by western blotting. β-actin was used as a control.

### Luciferase reporter assay

Luciferase reporter assays were performed as previously described [8]. Briefly, the luciferase reporter plasmids (TNF-α-wt and TNF-α-mt, DGCR8-wt and DGCR8-mt, nestin-wt and nestin-mt) were constructed by Gene-Chem. GSCs were seeded into 96-well plates at a density of 5 × 10^3^ cells per well and transfected with these plasmids for 48 h. Then the cells were lysed, and luciferase activity was measured using the Dual-Luciferase Reporter Assay System (Promega) according to the manufacturer’s instructions.

### Chromatin immunoprecipitation (ChIP) assays

According to the manufacturer’s instructions, ChIP assays were performed using the ChIP Assay Kit (Beyotime Biotechnology). Briefly, an anti-SPI1 antibody or normal rabbit IgG was used to immunoprecipitate the chromatin complexes. Then DNA was extracted and purified from the complexes and analyzed by qPCR. The primers for ChIP qPCR are listed in Table S5.

### RNA stability assay

RNA stability was measured as previously described [8]. Briefly, actinomycin D was used to block the de novo RNA synthesis, and GSCs were treated with 2 μg/ml actinomycin D (Act D, NobleRyder, China) for 24 h. Then total RNA was collected at 2, 4, 6, 8, 10 and 12 h, and circKPNB1 expression was detected by qPCR. The time required to reach 50% of the RNA levels before actinomycin D treatment was calculated and recognized as the half-life of circKPNB1.

### Protein stability evaluation

The proteasome inhibitor MG-132 (50 μM, Sigma-Aldrich) was added to GSCs for 6 h. Then the SPI1 proteins of GSCs were isolated and detected by western blotting. Besides, the cycloheximide (CHX) chase assay was also performed, and GSCs were treated with CHX (100 ng/ml, Sigma-Aldrich) for 0, 6, 12, 24, 36 h, followed by protein isolation and western blotting.

### Immunofluorescence

Immunofluorescence staining was performed as previously described [36]. Briefly, the GSCs were fixed, membrane permeabilized, antigen blocked and stained with the primary antibodies against SPI1 (1:100; Abcam), CD133 (1:100; Abcam) or nestin (1:100; Abcam) at 4 °C overnight. Then the fluorescein isothiocyanate-conjugated secondary antibodies (ProteinTech) were incubated, and the nuclei were counterstained with DAPI (Sigma-Aldrich). Finally, the GSCs were visualized using a laser scanning confocal microscope (Olympus).

### Enzyme-linked immunosorbent assay (ELISA)

The ELISA was performed using a commercial kit (Cusabio, Stratech, UK) to detect the concentration of TNF-α in the supernatant of GSCs medium as previously described [6]. The absorbance at 450 nm was detected using an ultraviolet spectrophotometer (ThermoFisher). All results were normalized to the protein concentration in the control group.

### Xenograft experiments

Female BALB/c nude mice (5–6 weeks old) were purchased from Shanghai Jihui Laboratory Animal Care Co., Ltd (Shanghai, China). All mice were bred in laminar flow cabinets under specific pathogen-free conditions in the Laboratory Animal Center of Shanghai Tenth People’s Hospital. With a stereotaxic apparatus, 5 × 10^4^ GSCs were injected orthotopically into the mouse brain at 2 mm lateral and 2 mm anterior to the bregma as previously described [6]. Each group contains five mice, and all mice were observed daily for signs of distress or death. The survival time of each mouse was calculated, and the tumor volume was calculated according to the formula: *V* = (*D* × *d*^2^)/2, where *D* represents the longest diameter and *d* represents the shortest diameter. All animal experiments were performed in accordance with the Animal Care Committee of Shanghai Tenth People’s Hospital.

### Immunohistochemistry (IHC)

IHC was performed using an immunohistochemical labeling kit (MaxVision Biotechnology, Fuzhou, Fujian, China) as previously described [6]. Briefly, paraffin-embedded sections of the intracranial tumor implantation specimens of nude mice were labeled with primary antibodies against SPI1 (1:100; Abcam), DGCR8 (1:100; Abcam), TNF-α (1:100; Abcam), nestin (1:100; Abcam) and Ki-67 (1:100; Abcam). The immunohistochemical results were evaluated according to the German immunohistochemical score (GIS) [38].

### Bioinformatics analysis

The basic information of circKPNB1 was obtained from circBase (http://www.circbase.org). The RBP targeting circKPNB1 was predicted from Cancer-Specific CircRNA Database (CSCD, http://gb.whu.edu.cn/CSCD/) and circInteractome (https://circinteractome.nia.nih.gov). The binding between circKPNB1 and proteins was predicted via CatRapid (http://service.tartaglialab.com/page/catrapid_group) and RNA-Protein Interaction Prediction (RPISeq, http://pridb.gdcb.iastate.edu/RPISeq/). Gene set enrichment analysis (GSEA, http:// www.broadinstitute.org/gsea/index.jsp) was used to detect the enrichment of signaling pathways based on the Cancer Genome Atlas (TCGA, http:// cancergenome.nih.gov) and the Chinese Glioma Genome Atlas (CGGA, http://www.cgga.org.cn).

### Statistical analysis

Statistical analysis was performed using SPSS 23.0 software (IBM, Armonk, NY, USA) or GraphPad Prism 8.0 (GraphPad Software Inc, San Diego, C.A, USA). All experiments were repeated at least three times, and the results are presented as the mean ± SD. The chi-square test, two-tailed Student’s *t*-test, and one-way analysis of variance were used to compare the statistical significance among different groups. The survival rates for humans and mice were analyzed using the log-rank test and Kaplan–Meier analysis. Receiver operating characteristic (ROC) curves were made using Xiantao (www.xiantao.love). Statistical significance was defined when the two-tailed *P* values were <0.05.

## Supplementary information

Figure S1

Figure S2

Figure S3

Figure S4

Supplementary figure legends

Table S1

Table S2

Table S3

Table S4

Table S5

Original western blots

aj-checklist

## Data Availability

The analyzed data sets generated during the present study are available from the corresponding author on reasonable request.
