# Supplementary figures and images for: CircKPNB1 mediates a positive feedback loop and promotes the malignant phenotypes of GSCs via TNF-α/NF-κB signaling
Source: Cell Death Dis. 2022 Aug 9;13(8):697. doi: 10.1038/s41419-022-05149-1 (PMC9363451; doi:10.1038/s41419-022-05149-1)

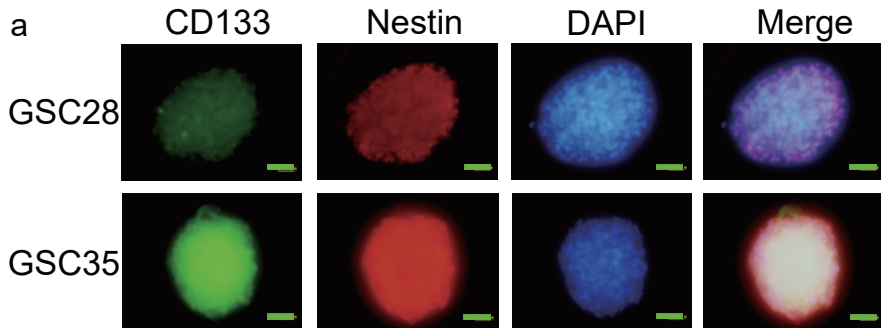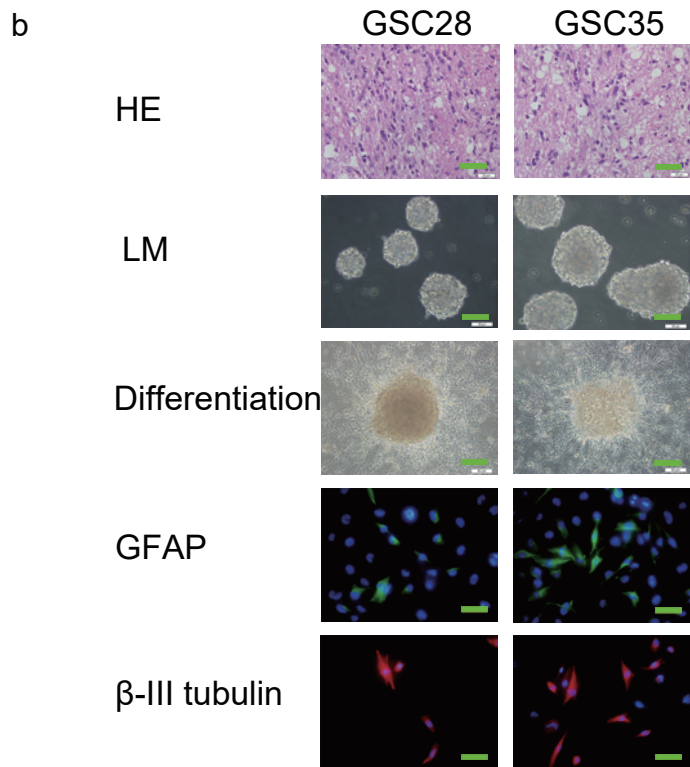

Supplement: Supplementary file 1 — Figure S1 [file 41419_2022_5149_MOESM1_ESM.pdf]

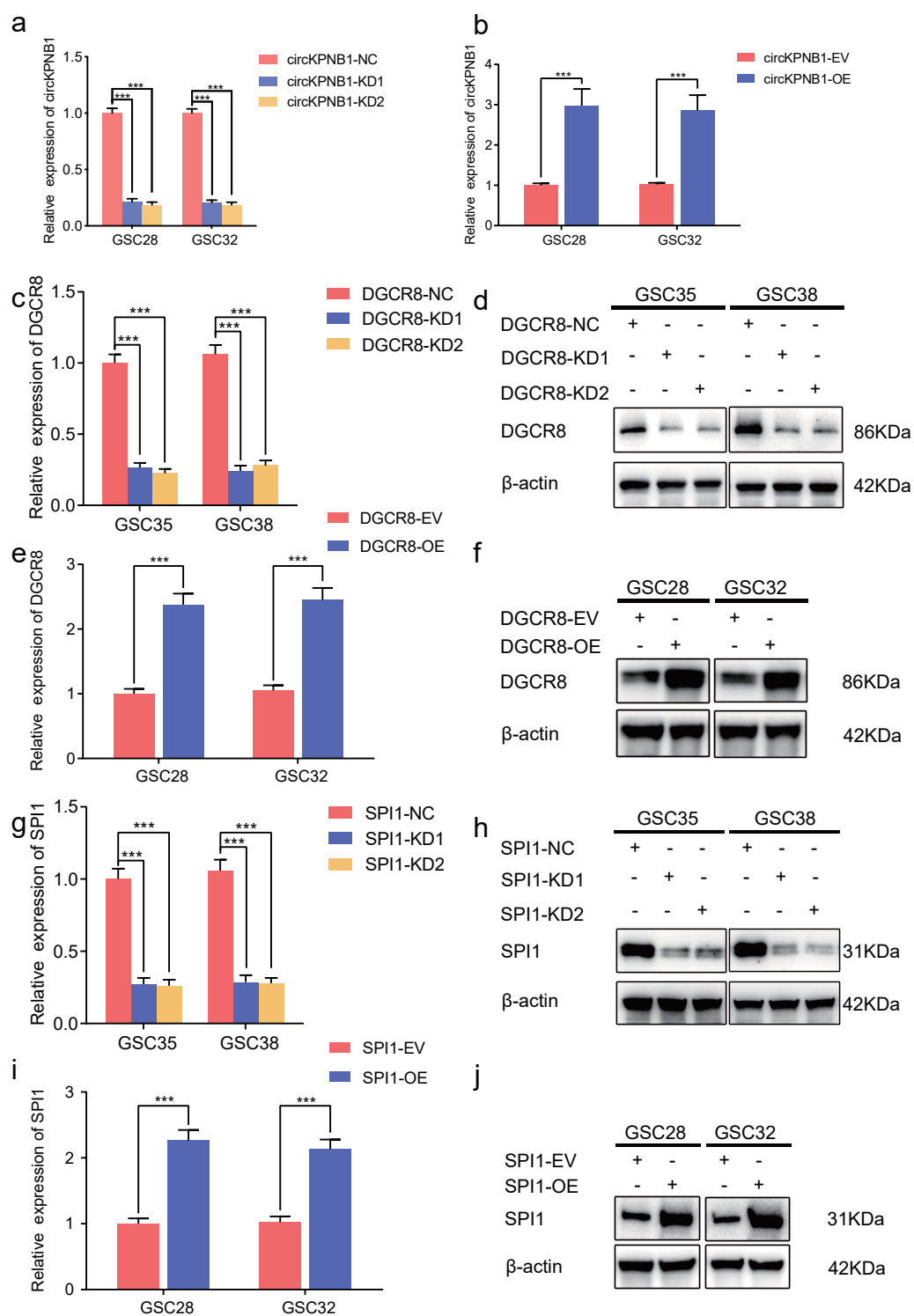

Supplement: Supplementary file 2 — Figure S2 [file 41419_2022_5149_MOESM2_ESM.pdf]

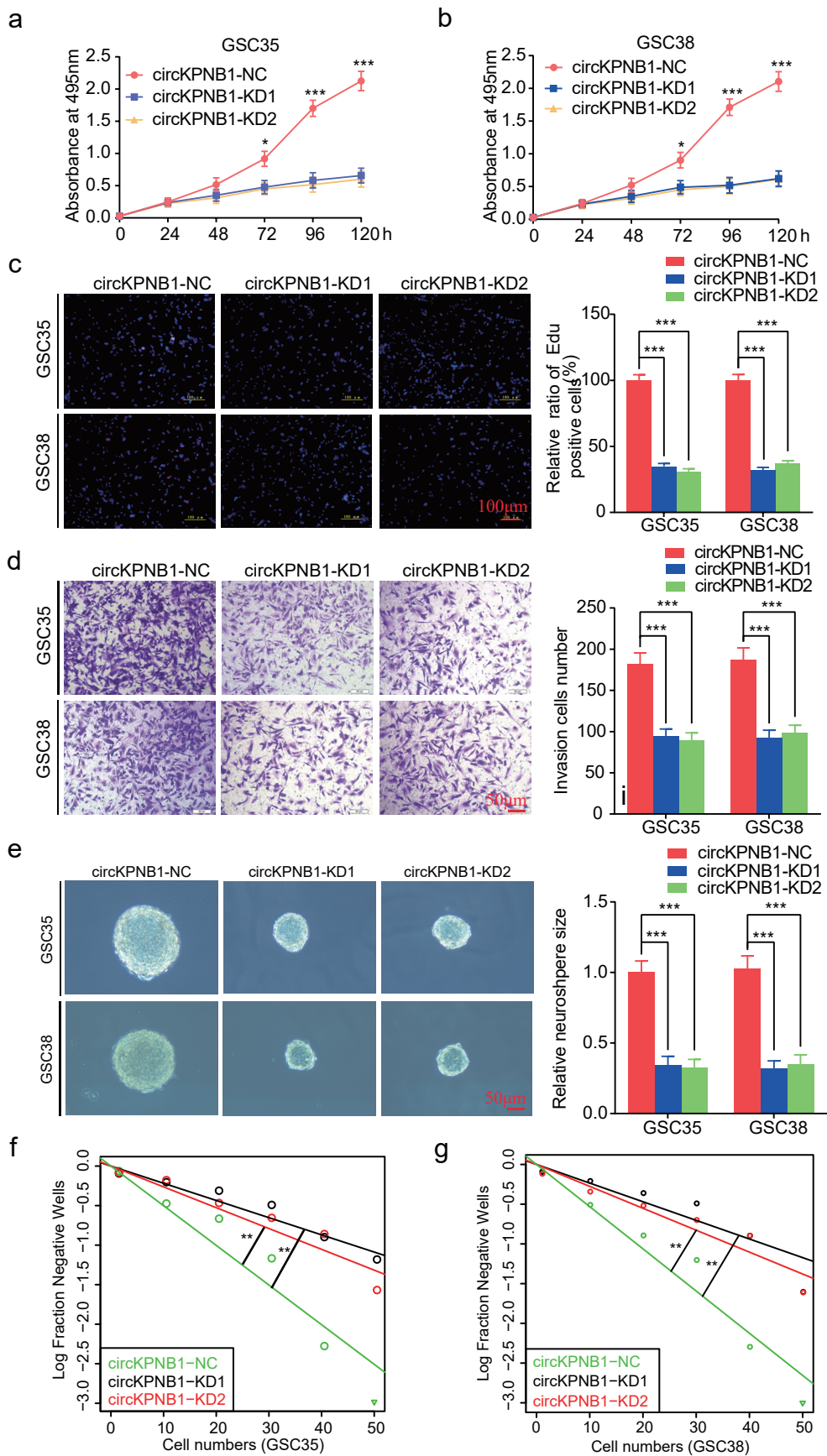

Supplement: Supplementary file 3 — Figure S3 [file 41419_2022_5149_MOESM3_ESM.pdf]

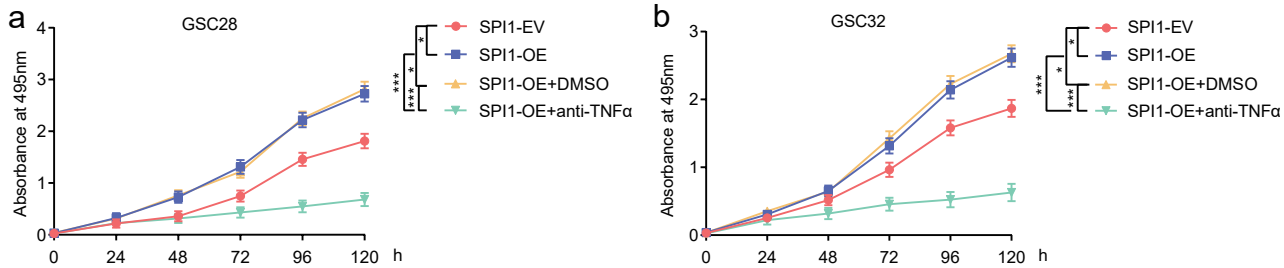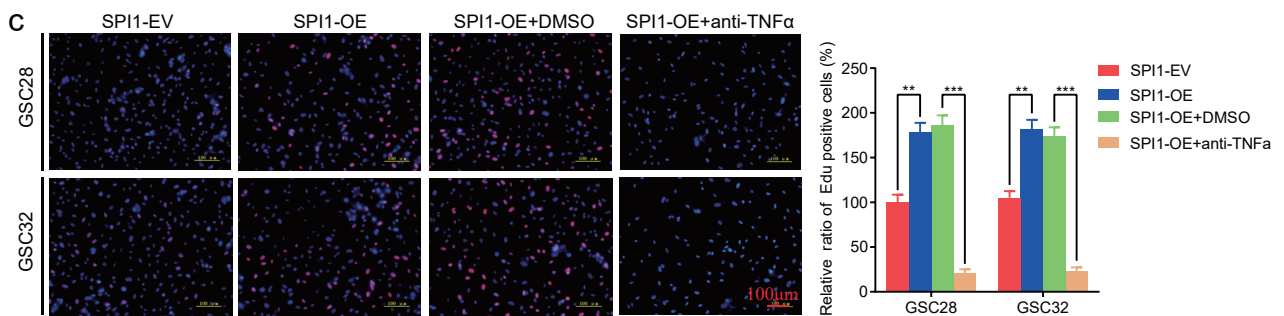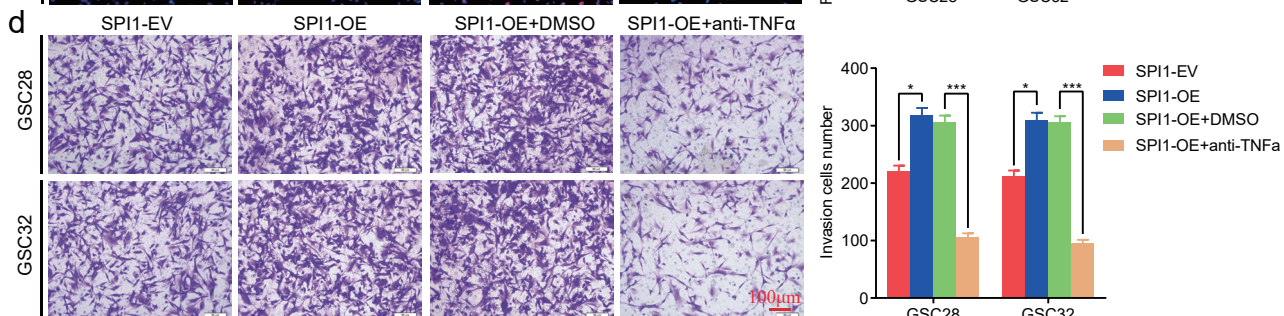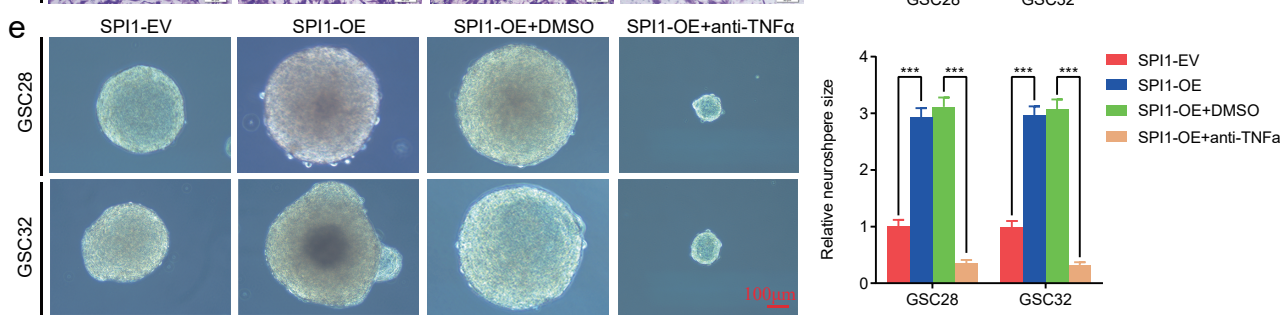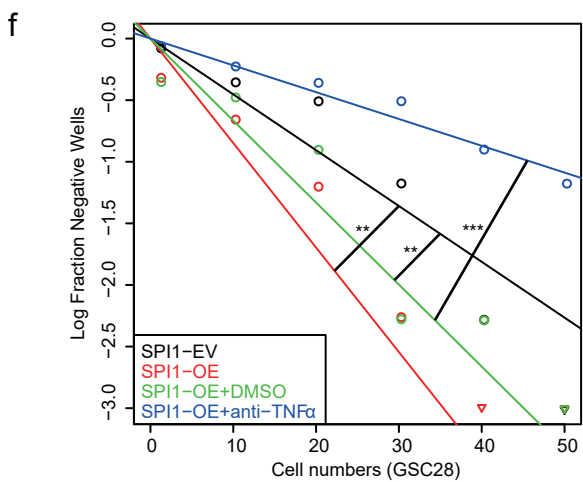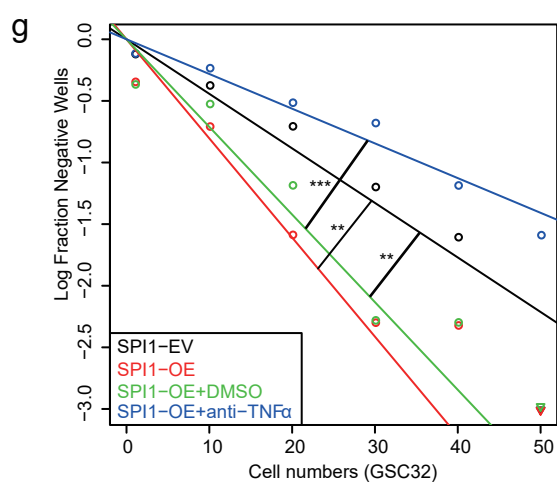

Supplement: Supplementary file 4 — Figure S4 [file 41419_2022_5149_MOESM4_ESM.pdf]

Figure 1 d, e


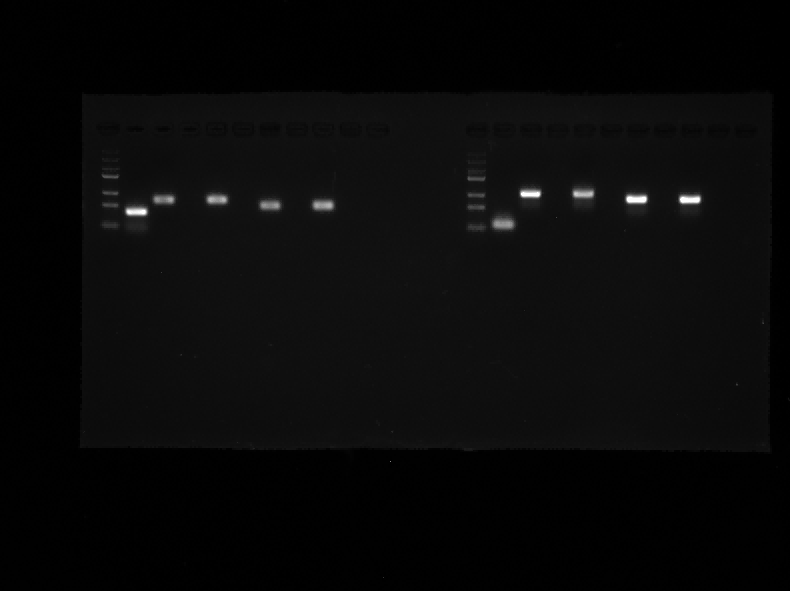


Figure 3

F


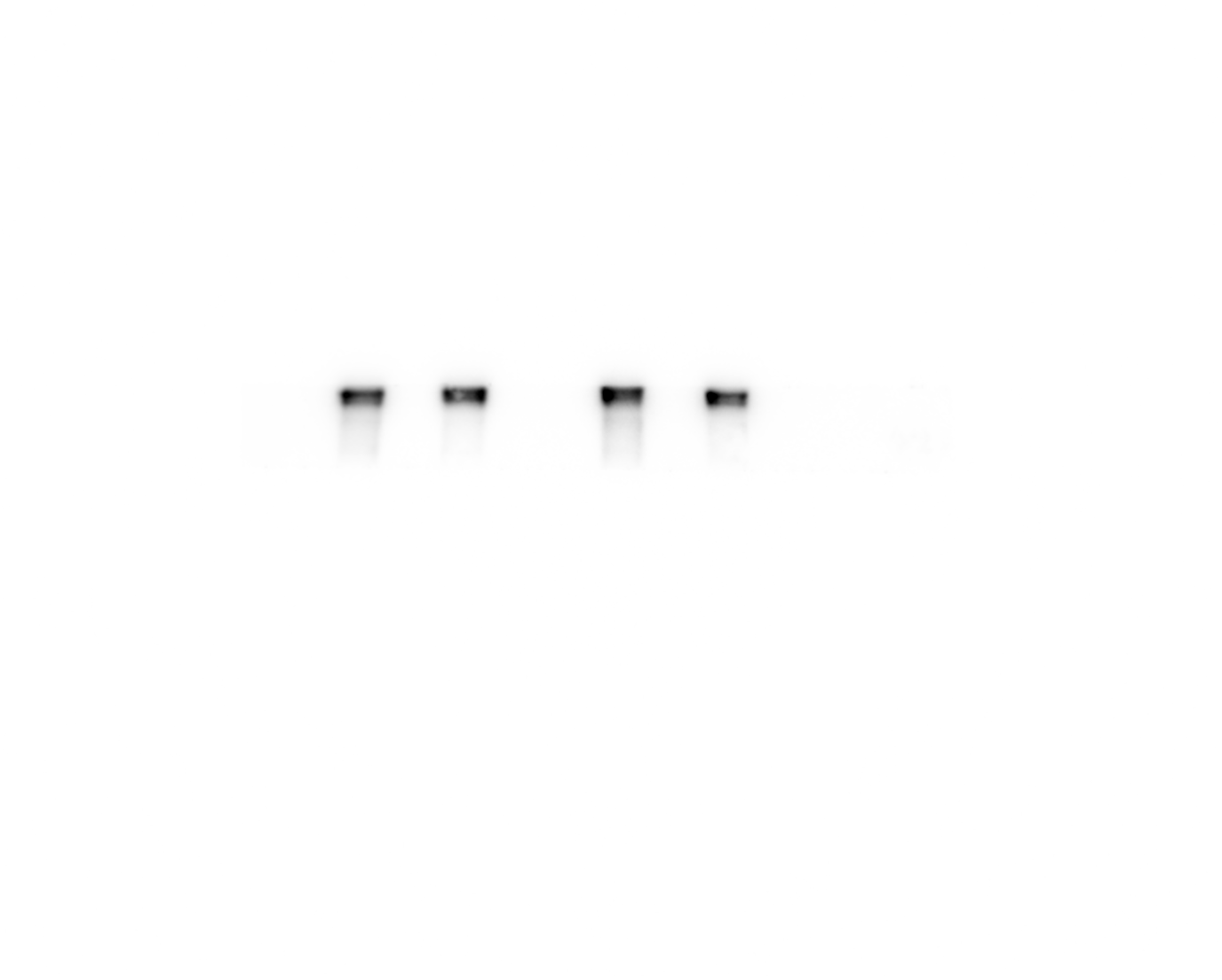

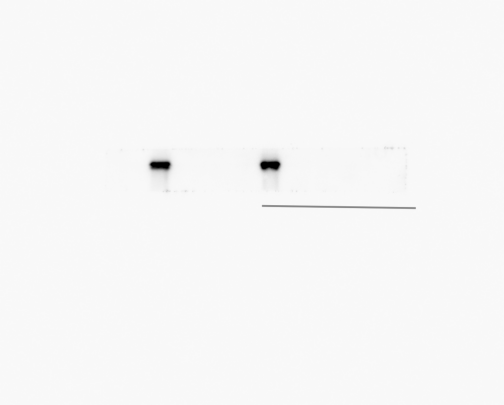


g


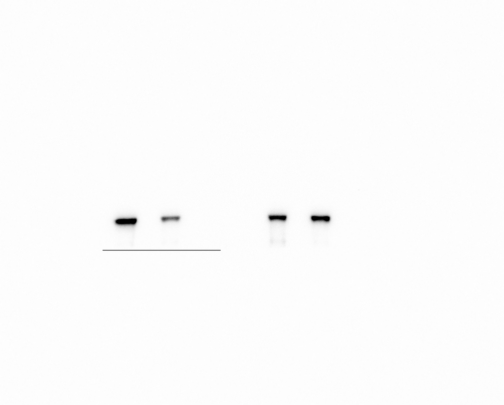

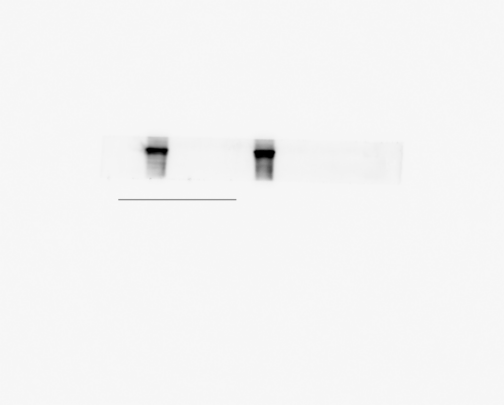


i


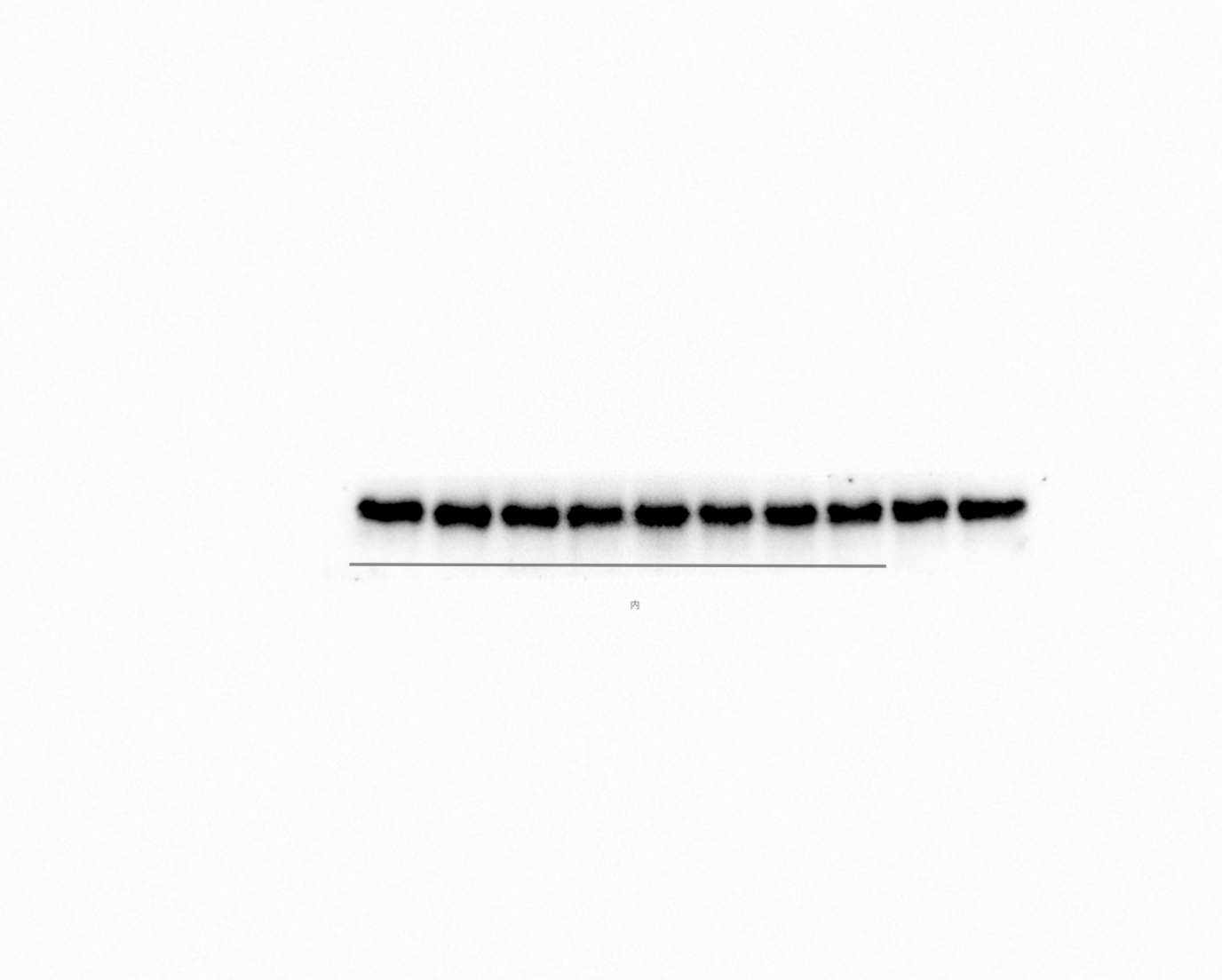

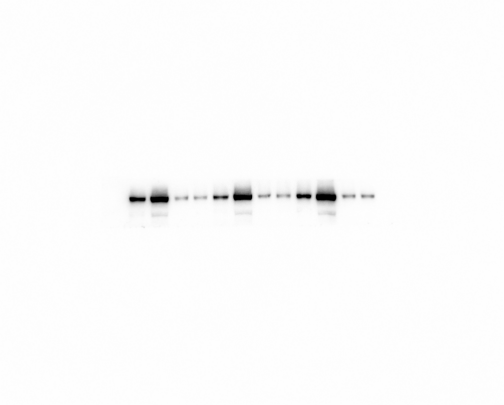


j


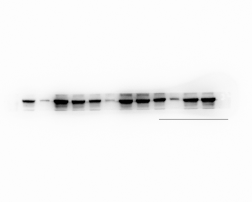

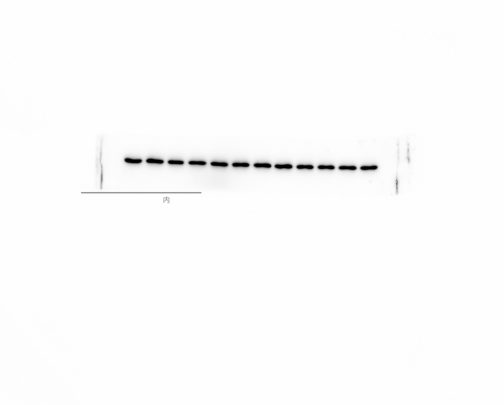


K


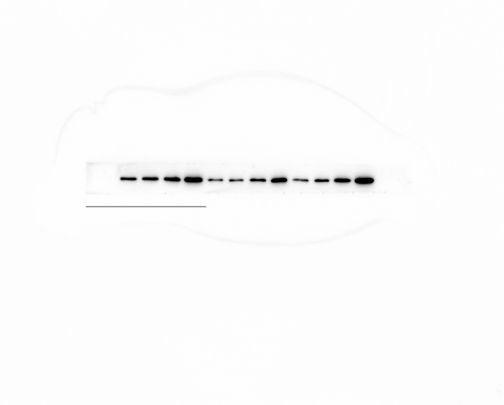

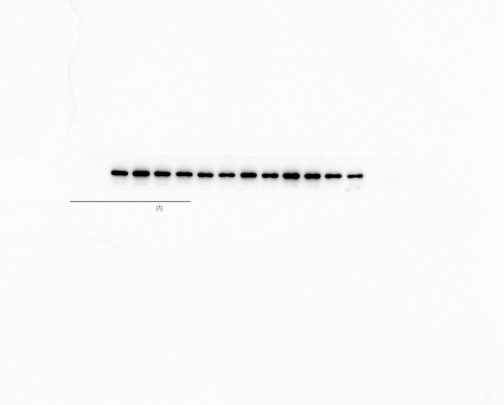


L


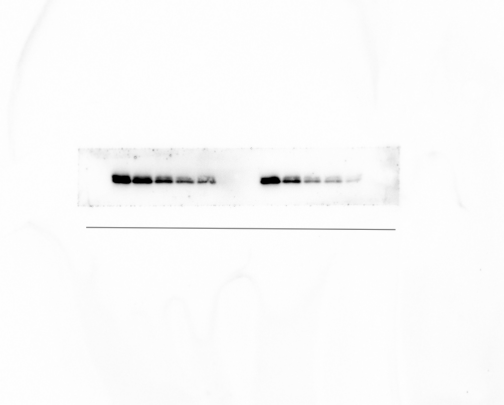

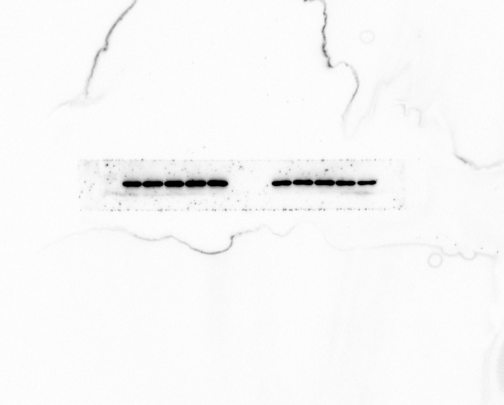


N


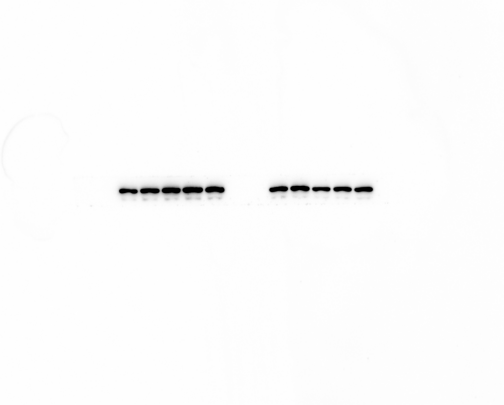

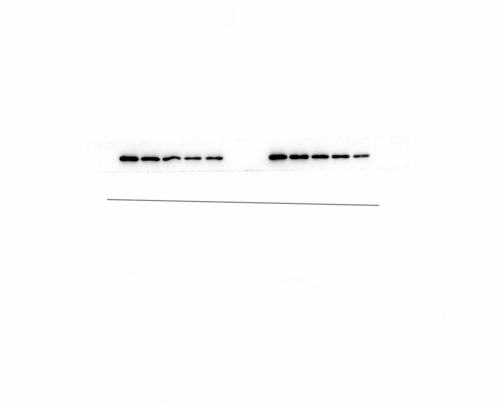


O


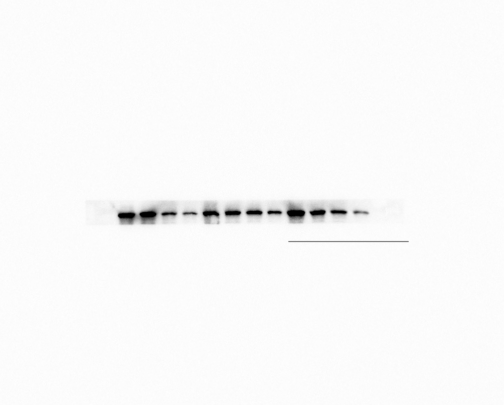

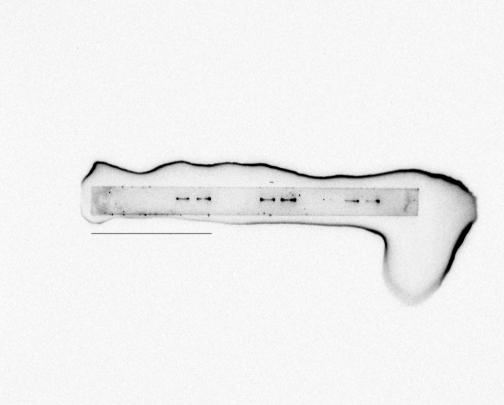

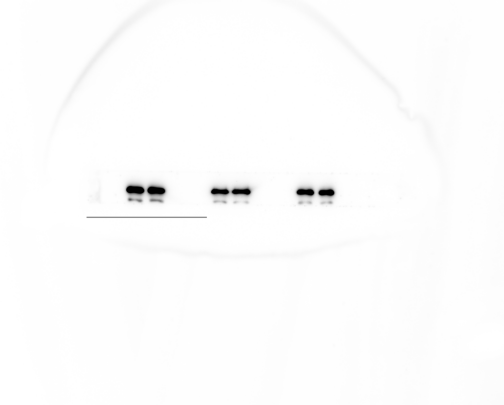


P


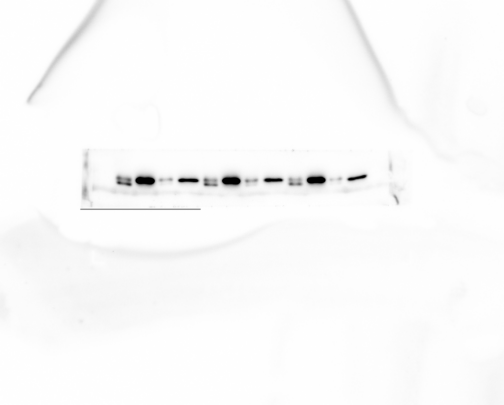

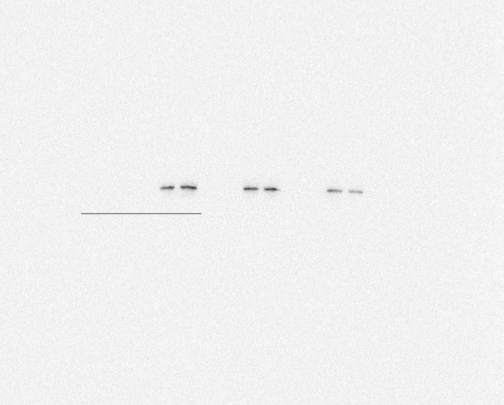


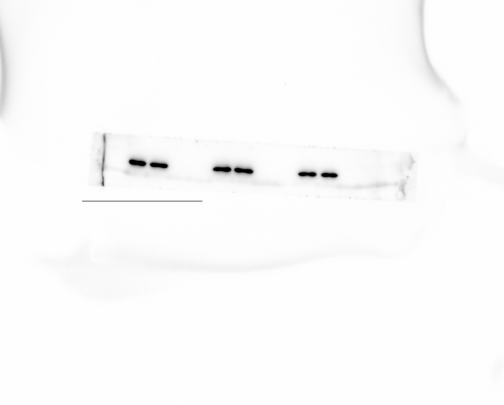


Figure 5

b, c


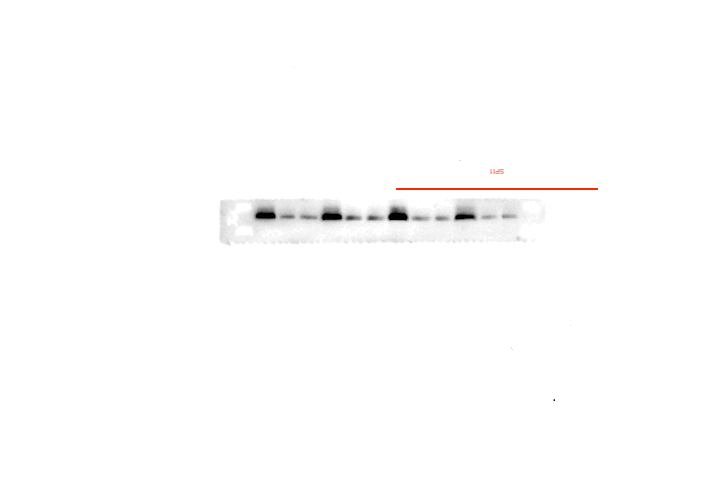


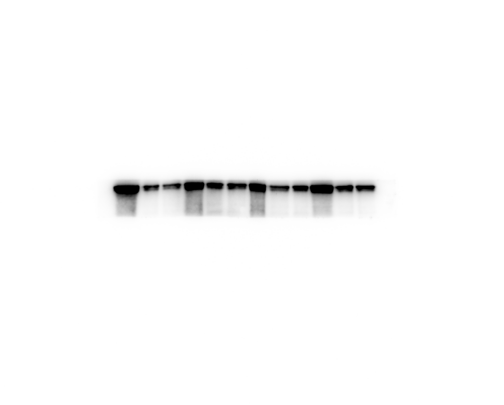

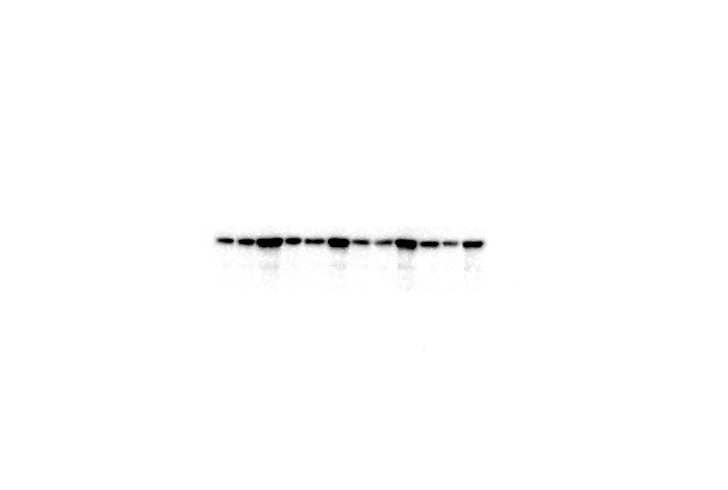


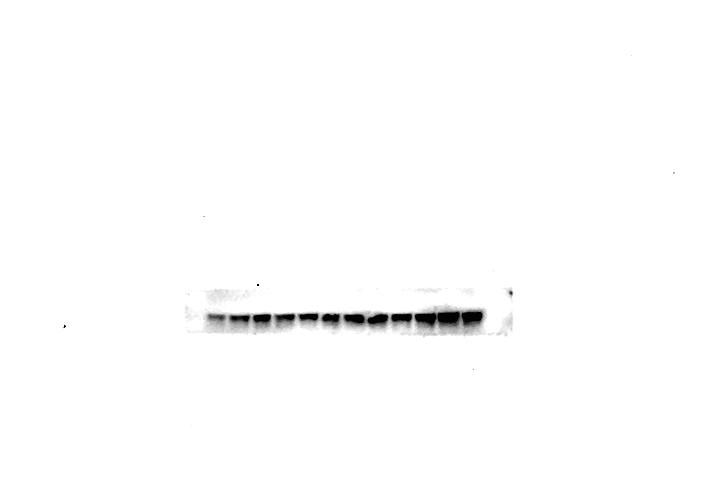


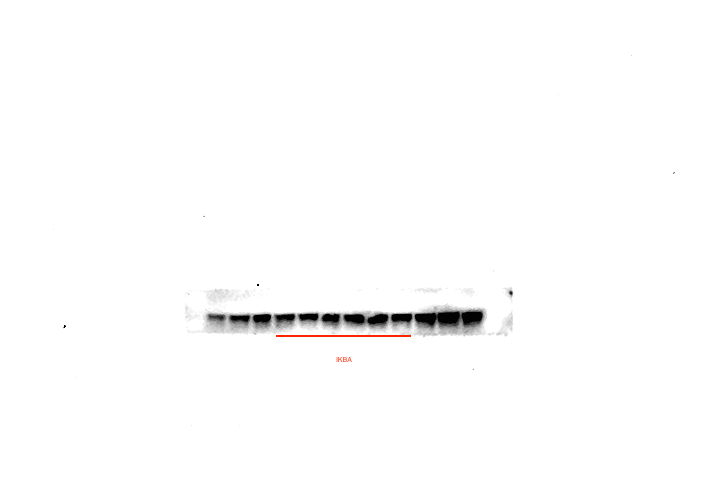


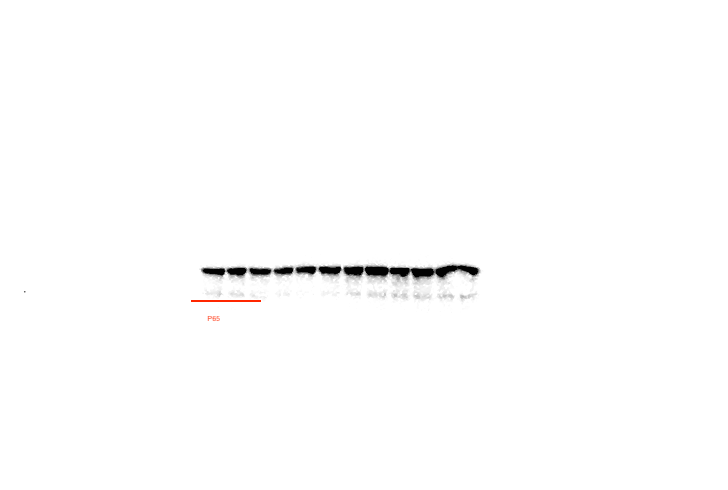


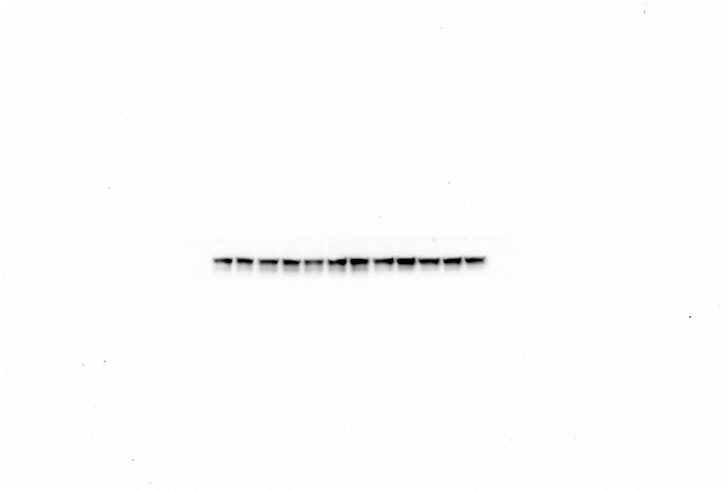


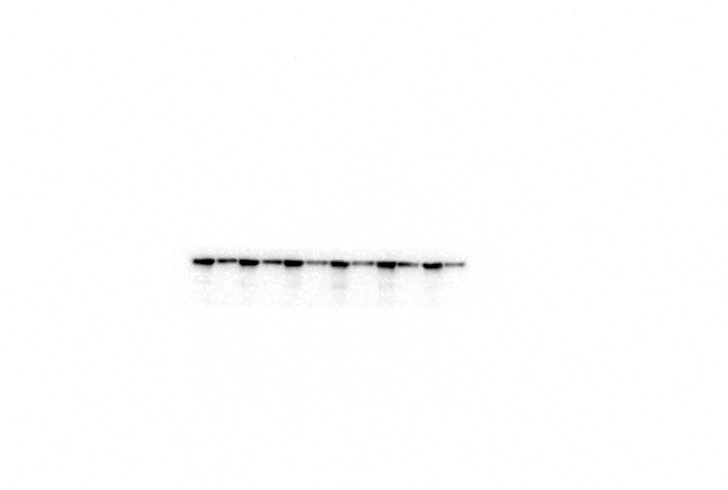


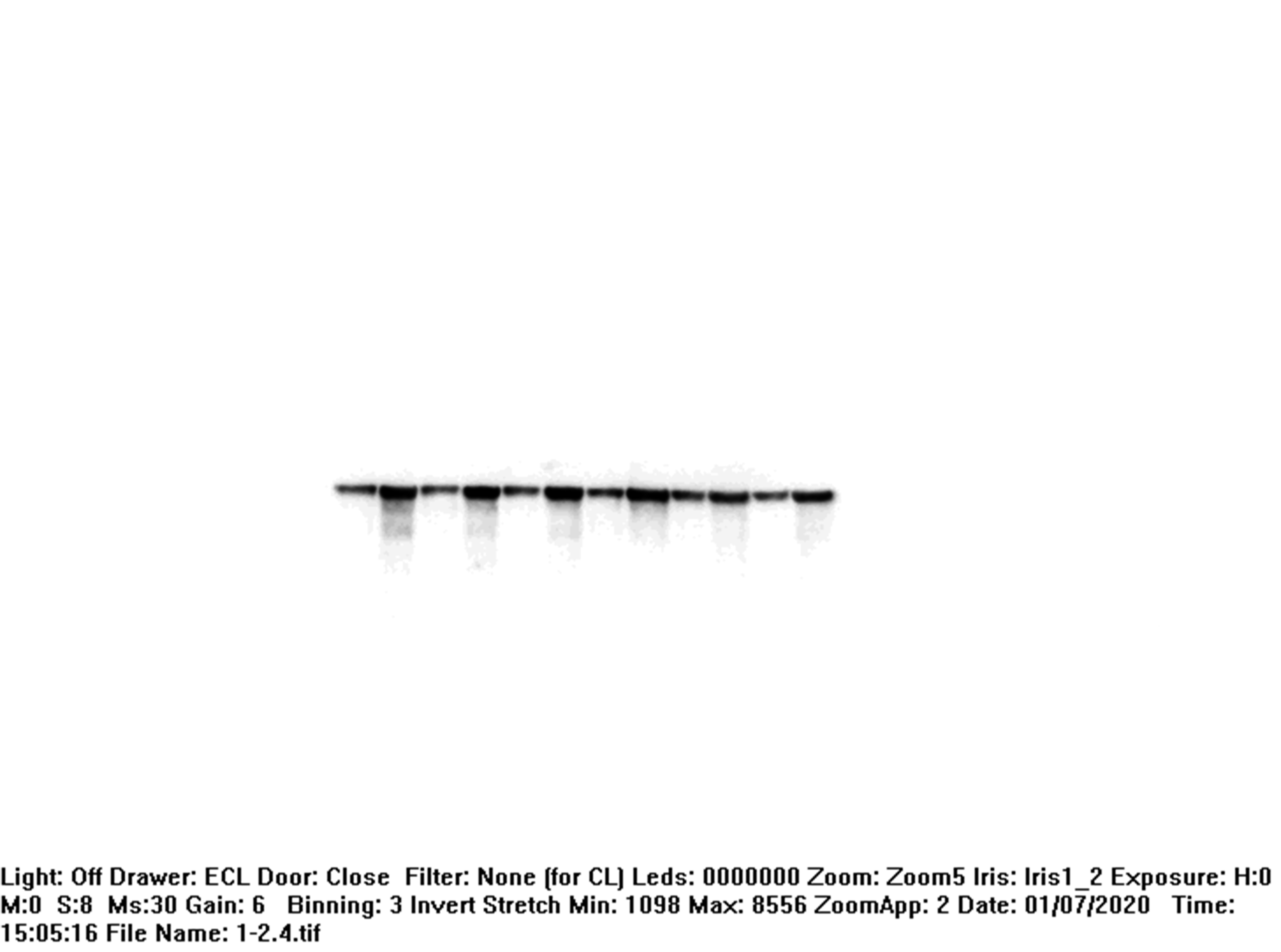


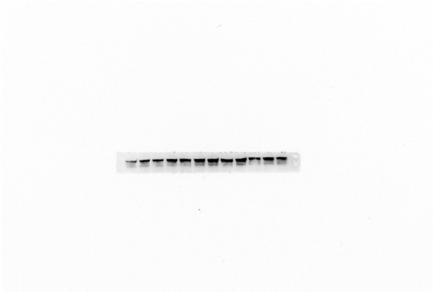

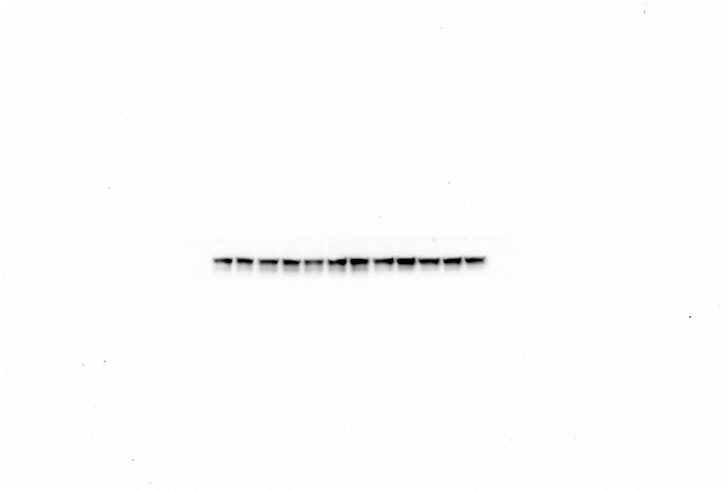


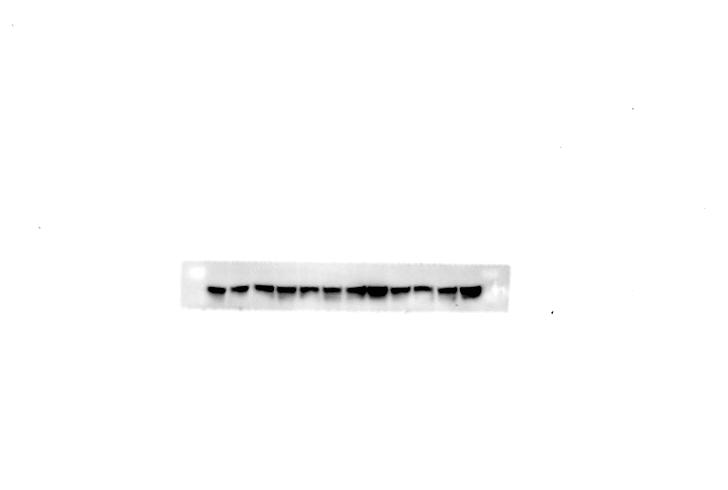


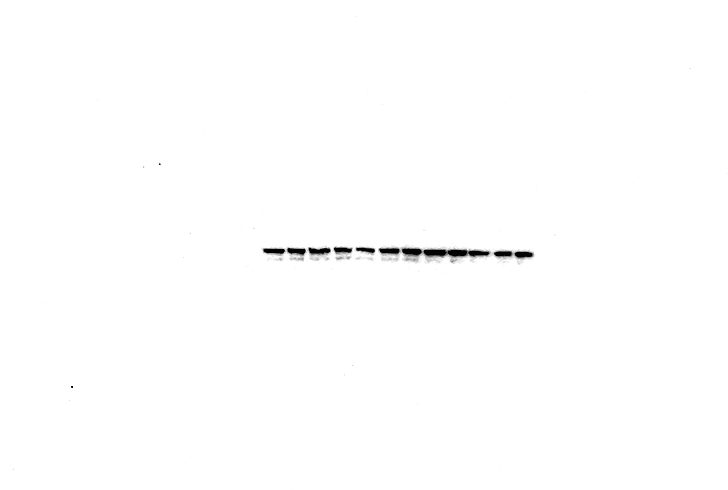


Figure 6

I


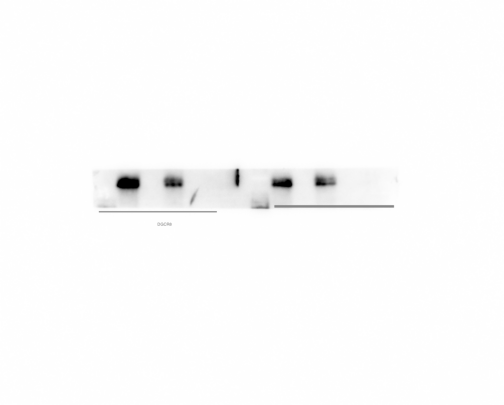

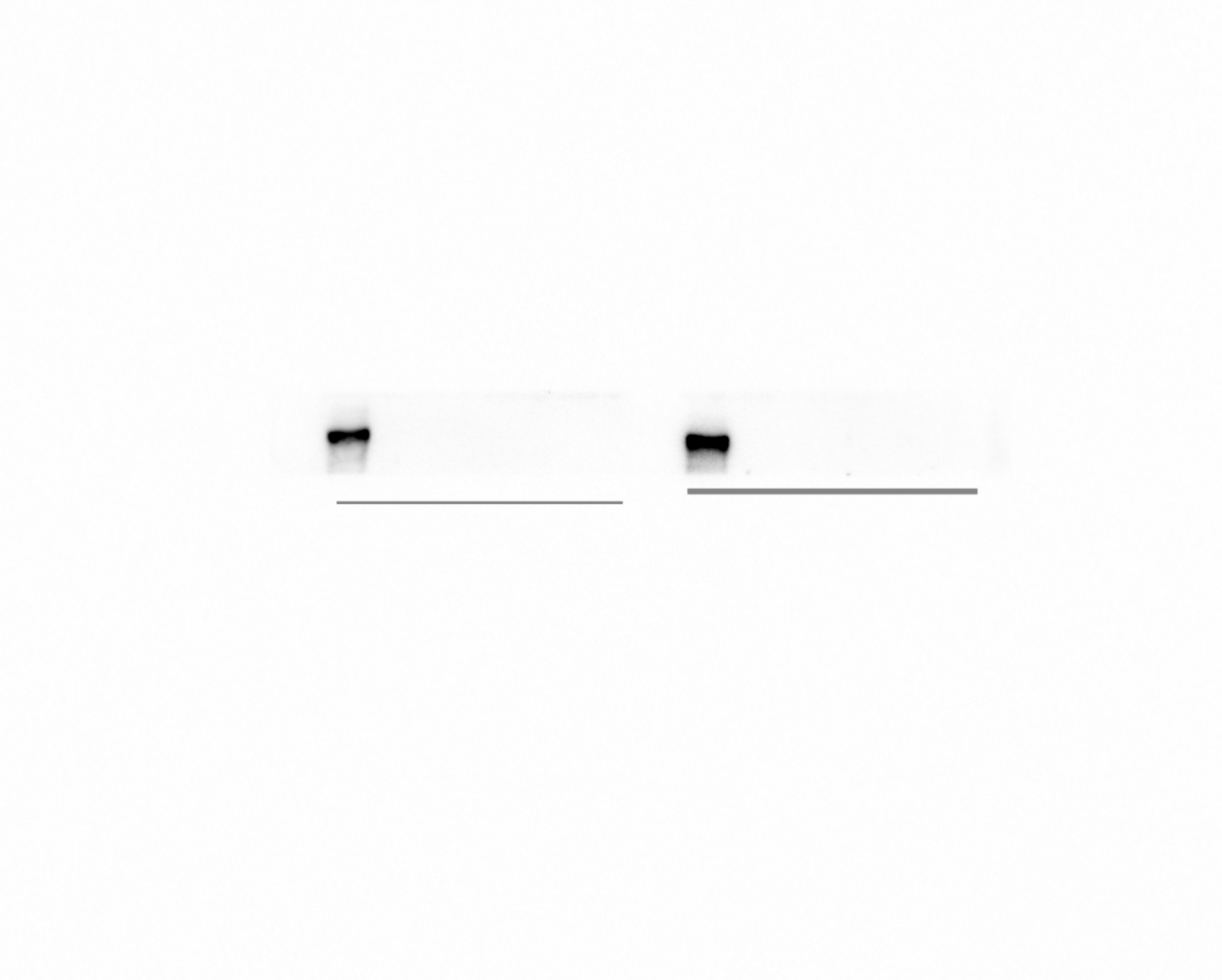


J


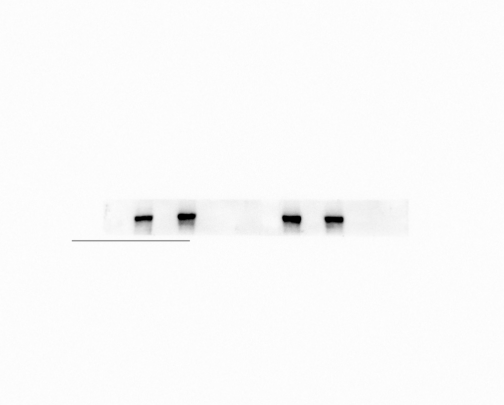

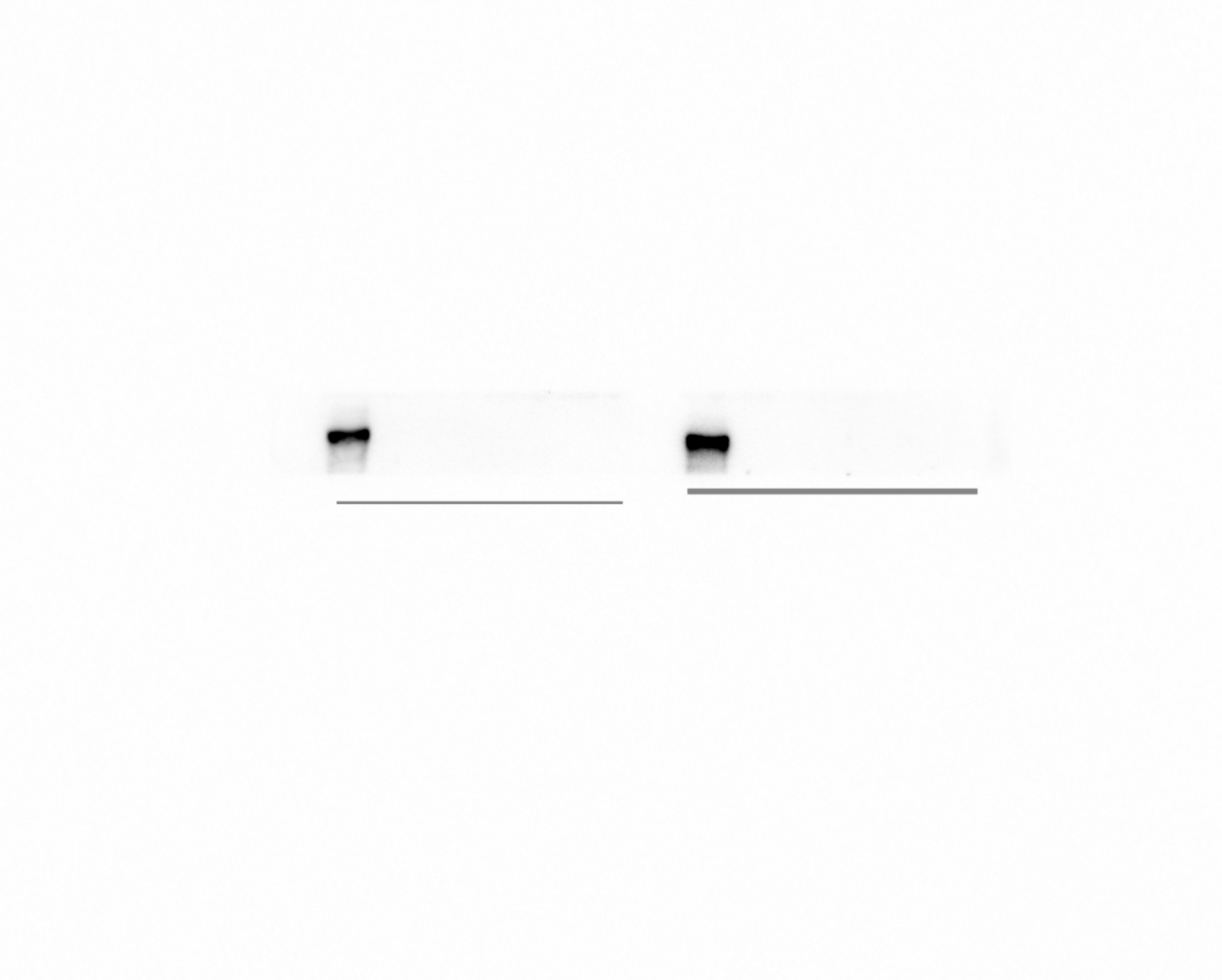


Figure 7

E


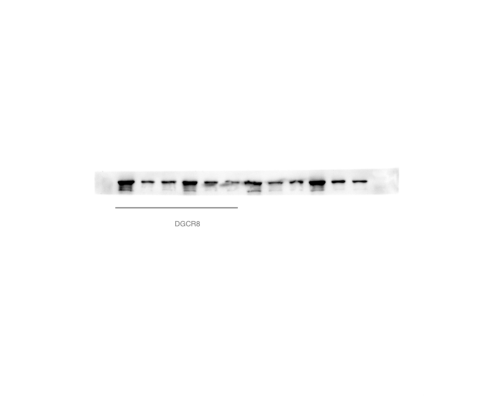

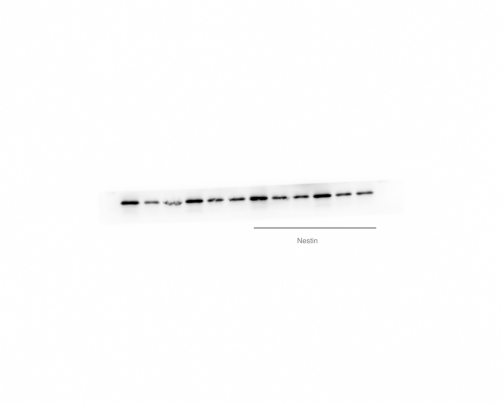

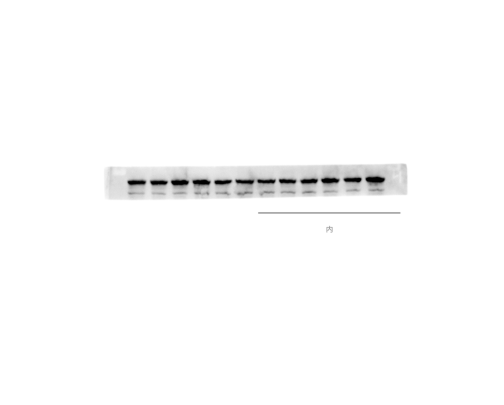


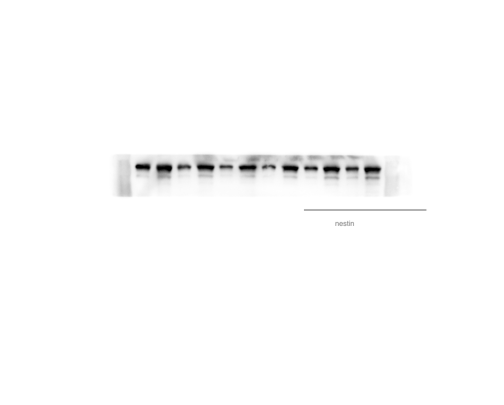

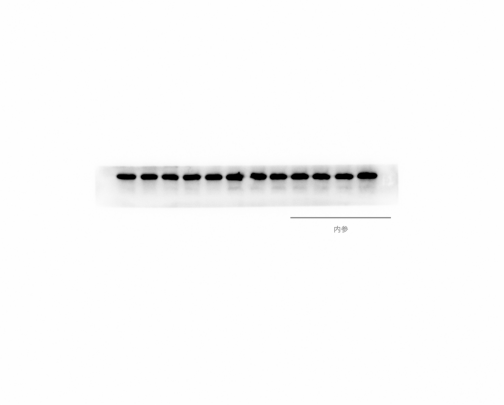

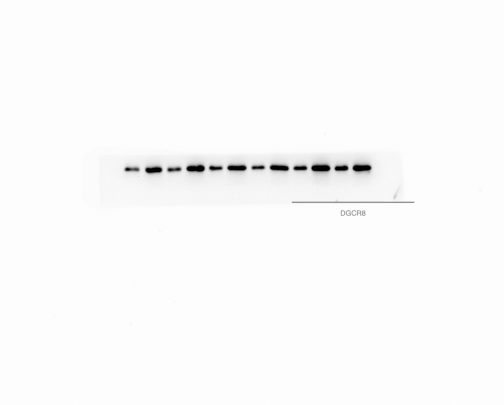


U


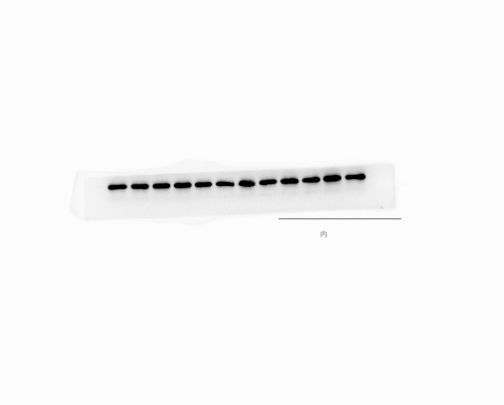

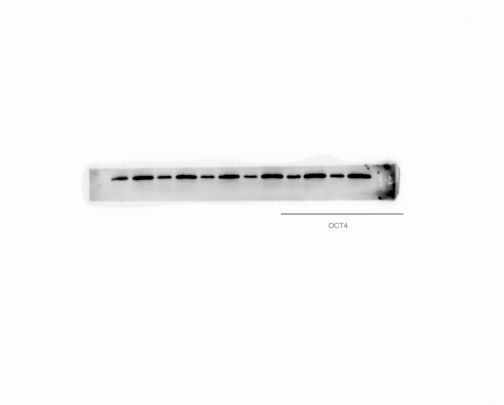

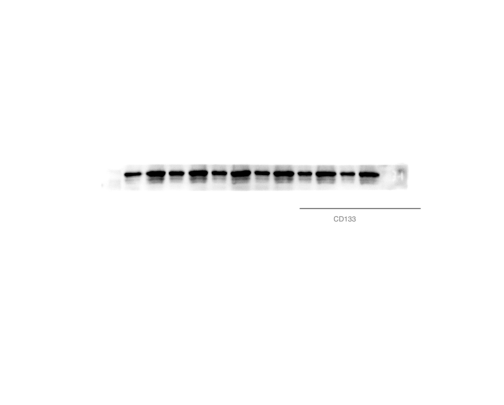

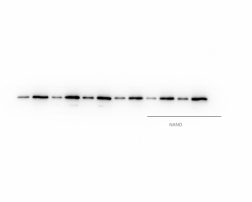

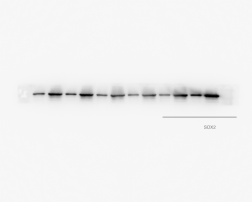


V


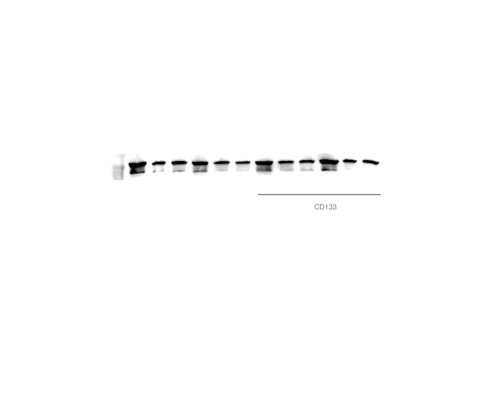

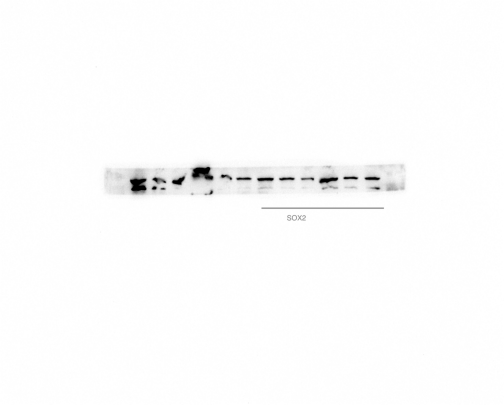

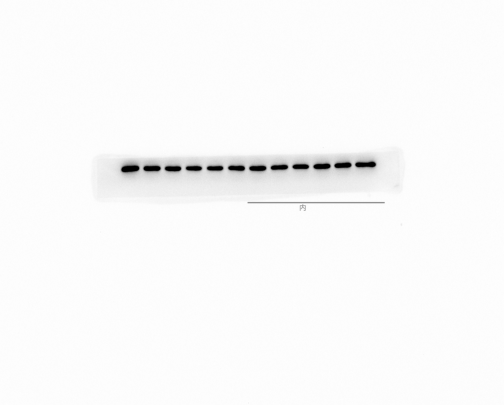

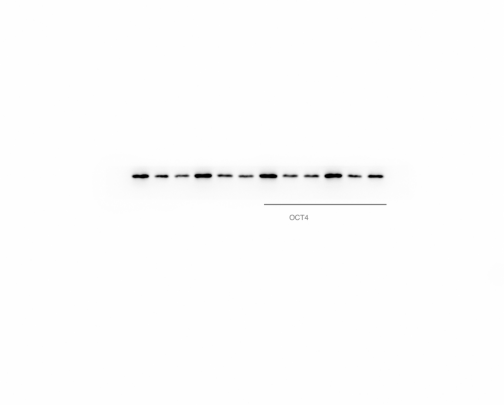

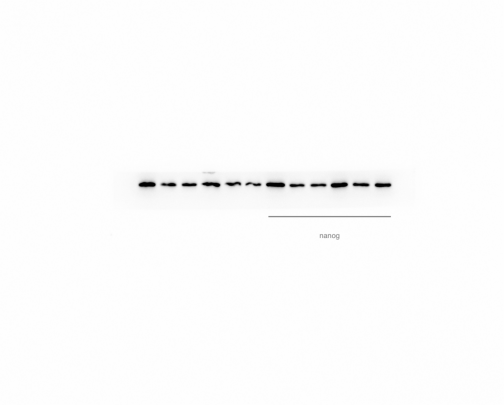


Supplementary figure 2


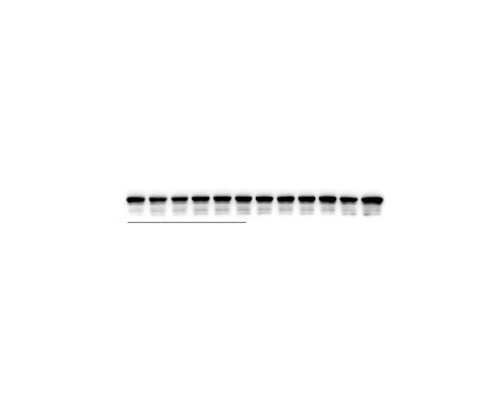

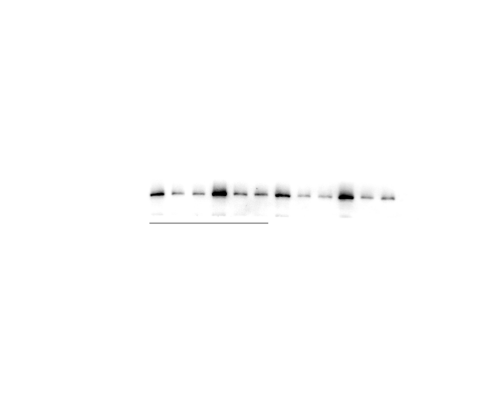

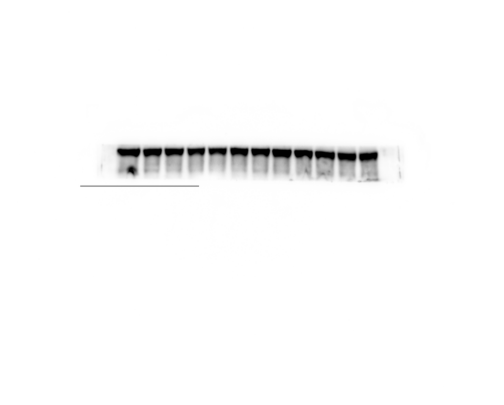

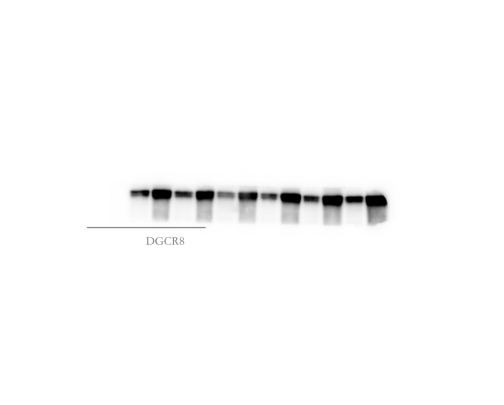

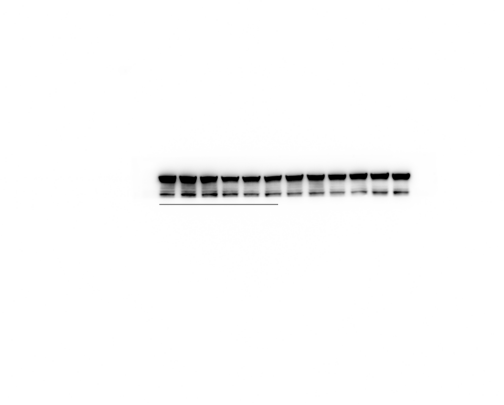

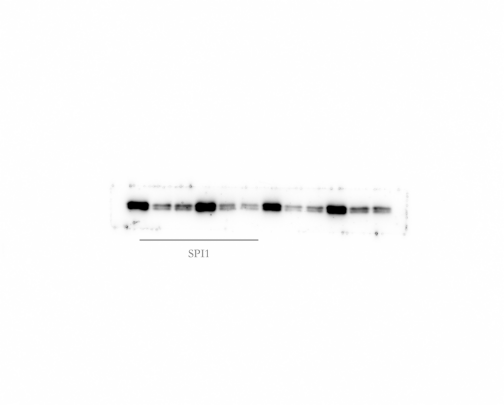

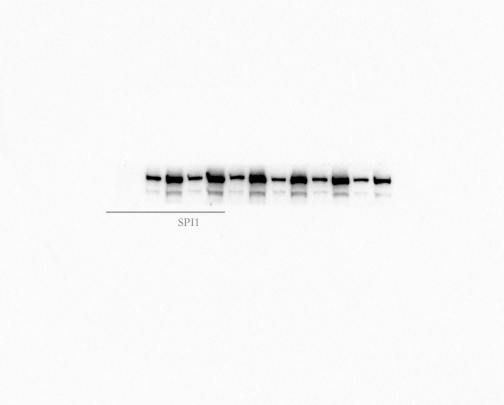

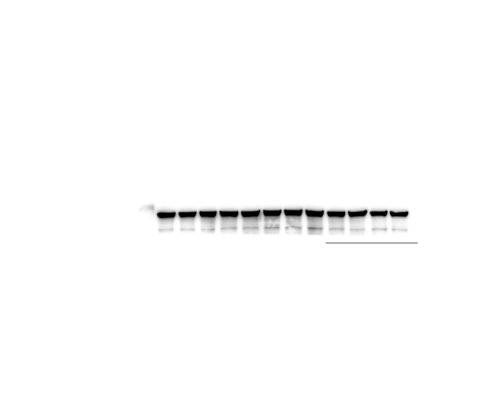

Supplement: Supplementary file 11 — Original western blots [file 41419_2022_5149_MOESM11_ESM.docx]
