# Supplementary material for: CircKPNB1 mediates a positive feedback loop and promotes the malignant phenotypes of GSCs via TNF-α/NF-κB signaling
Source: Cell Death Dis. 2022 Aug 9;13(8):697. doi: 10.1038/s41419-022-05149-1 (PMC9363451; doi:10.1038/s41419-022-05149-1)
Supplement: Supplementary file 5 — Supplementary figure legends [file 41419_2022_5149_MOESM5_ESM.docx]

**Supplementary figure 1 Isolation and validation of patient-derived glioma stem cells (GSCs).**

a: Immunofluorescence staining showing GSCs' stem cell markers (CD133 and nestin). Scale bar = 20 μm.

b: The GBM specimens used to isolate GSC28 and GSC32 were shown by hematoxylin-eosin (HE) staining. Scale bar = 50 μm. Representative microphotographs showing the morphological character of GSCs. Scale bar = 50 μm. Representative microphotographs showing the morphological changes after GSCs differentiated. Scale bar = 50 μm. Immunofluorescence staining showing GFAP and β III tubulin expression after GSCs differentiated. Scale bar = 50 μm.

**Supplementary figure 2 The expression of circKNPB1, SPI1, and DGCR8 in GSCs after lentiviral-based transfection.**

a, b: qPCR showing circKNPB1 expression after circKNPB1 knockdown (a) or overexpression (b) in GSC28 and GSC32.

c, e: qPCR showing DGCR8 expression after DGCR8 knockdown (c) or overexpression (e) in GSC28 and GSC32.

d, f: Western blotting showing DGCR8 expression after DGCR8 knockdown (d) or overexpression (f) in GSC28 and GSC32.

g, i: qPCR showing SPI1 expression after SPI1 knockdown (g) or overexpression (i) in GSC28 and GSC32.

h, j: Western blotting showing DGCR8 expression after DGCR8 knockdown (h) or overexpression (j) in GSC28 and GSC32.

All data are expressed as the mean ± SD (three independent experiments). *p < 0.05; **p < 0.01; ***p < 0.001.

**Supplementary figure 3 CircKPNB1 knockdown inhibits the malignant phenotype of GSCs in vitro.**

a, b: MTS assays showing the cell viabilities changes in circKNPB1 silenced GSC38 and GSC35.

c: EDU assays showing the proliferation changes in circKNPB1 silenced GSC38 and GSC35. Scale bar = 100 μm.

d: Transwell assays showing the change of invasion cell numbers after circKNPB1 silenced in GSC38 and GSC35. Scale bar = 50 μm.

e: Neuroshperes formation assays showing the neurospheres formation abilities after circKNPB1 silenced in GSC28 and GSC32. Scale bar = 50 μm.

f, g: Limiting dilution assays showing the self-renewing capacity of GSC4D circKNPB1 silenced GSC28 and GSC32.

All data are expressed as the mean ± SD (three independent experiments). *p < 0.05; **p < 0.01; ***p < 0.001.

**supplementary figure 4 SPI1 can promote the malignant phenotype of GSCs via TNFα mediated NF-κb signaling.**

a, b: MTS assays showing the cell viability changes in SPI1 overexpressed GSCs following with anti-TNF-α treatment.

c: EDU assays showing the proliferation changes in SPI1 overexpressed GSCs with anti-TNF-α treatment. Scale bar = 100 μm.

d: Transwell assays showing the change of invasion cell numbers in SPI1 overexpressed GSCs following with anti-TNF-α treatment. Scale bar = 50 μm.

e: Neuroshperes formation assays showing the neurospheres formation abilities of SPI1 overexpressed GSCs following with anti-TNF-α treatment. Scale bar = 50 μm.

f, g: Limiting dilution assays showing the self-renewing capacity of SPI1 overexpressed GSCs following with anti-TNF-α treatment.

All data are expressed as the mean ± SD (three independent experiments). *p < 0.05; **p < 0.01; ***p < 0.001.
