# Supplementary material for: CircKPNB1 mediates a positive feedback loop and promotes the malignant phenotypes of GSCs via TNF-α/NF-κB signaling
Source: Cell Death Dis. 2022 Aug 9;13(8):697. doi: 10.1038/s41419-022-05149-1 (PMC9363451; doi:10.1038/s41419-022-05149-1)
Supplement: Supplementary file 6 — Table S1 [file 41419_2022_5149_MOESM6_ESM.docx]

**Supplementary Table 1.** Relationship of CircKPNB1 expression to clinical features of glioma patients

| **Clinical features** | | **Samples**  **(*n* = 70)** | **CircKPNB1 expression*** | | ***P* value** |
| --- | --- | --- | --- | --- | --- |
|  |  |  | **Low (*n* = 35)** | **High (*n* = 35)** |  |
| **Sex** | Male | 33 | 15 | 18 | *P*=0.473 |
|  | Female | 37 | 20 | 17 |  |
| **Age** | ≤ 50 | 29 | 18 | 11 | *P*=0.089 |
|  | > 50 | 41 | 17 | 24 |  |
| **WHO grade** | Ⅱ | 20 | 13 | 7 | ***P*=0.022** |
|  | Ⅲ | 25 | 15 | 10 |  |
|  | Ⅳ | 25 | 7 | 18 |  |
| **IDH**  **status** | Wild | 33 | 10 | 23 | ***P=*0.002** |
|  | Mutant | 37 | 25 | 12 |  |
| **1p/19q status** | Codeletion | 36 | 23 | 13 | ***P*=0.017** |
|  | Non-codeletion | 34 | 12 | 22 |  |
| **H3F3A status** | Wild | 39 | 25 | 14 | ***P*=0.008** |
|  | Mutant | 31 | 10 | 21 |  |
| **MGMT**  **status** | Methylation | 41 | 25 | 16 | ***P*=0.029** |
|  | Unmethylation | 29 | 10 | 19 |  |

*: CircKPNB1 expression was detected by qRT-PCR and ranked from low to high. The high expression of CircKPNB1 was defined as the expression level higher than the median expression level of CircKPNB1.
