# Supplementary material for: CircKPNB1 mediates a positive feedback loop and promotes the malignant phenotypes of GSCs via TNF-α/NF-κB signaling
Source: Cell Death Dis. 2022 Aug 9;13(8):697. doi: 10.1038/s41419-022-05149-1 (PMC9363451; doi:10.1038/s41419-022-05149-1)
Supplement: Supplementary file 7 — Table S2 [file 41419_2022_5149_MOESM7_ESM.docx]

Supplementary Table 2. **Clinical information of the primary glioma stem-like cells.**

|  | GSC28 | GSC31 | GSC32 | GSC35 | GSC38 | GSC39 |
| --- | --- | --- | --- | --- | --- | --- |
| Gender | Female | Female | Male | Female | Male | Female |
| Age | 56 years old | 51 years old | 55 years old | 57 years old | 56 years old | 54 years old |
| Location | Left frontal lobe | Right occipital lobe | Left frontal lobe | Right insula | Right frontal lobe | Left occipital lobe |
| Pathological diagnosis | Glioblastoma | Glioblastoma | Glioblastoma | Glioblastoma | Glioblastoma | Glioblastoma |
| WHO grade | IV | IV | IV | IV | IV | IV |
| Ki-67 | 25% (+) | 30% (+) | 25% (+) | 50% (+) | 50% (+) | 30% (+) |
| IDH status | Wild | Wild | Wild | Wild | Wild | Wild |
| 1p/19q status | Non-codeletion | Non-codeletion | Non-codeletion | Non-codeletion | Non-codeletion | Non-codeletion |
| H3F3A status | Mutant | Mutant | Mutant | Mutant | Mutant | Mutant |
| MGMT status | Unmethylation | Unmethylation | Unmethylation | Unmethylation | Unmethylation | Unmethylation |
