# Supplementary material for: CircKPNB1 mediates a positive feedback loop and promotes the malignant phenotypes of GSCs via TNF-α/NF-κB signaling
Source: Cell Death Dis. 2022 Aug 9;13(8):697. doi: 10.1038/s41419-022-05149-1 (PMC9363451; doi:10.1038/s41419-022-05149-1)
Supplement: Supplementary file 8 — Table S3 [file 41419_2022_5149_MOESM8_ESM.docx]

| **Primer** | **Forward (5’-3’)** | **Reverse (5’-3’)** |
| --- | --- | --- |
| circKPNB1-KD1 | UUUCAUUGCUUCGAUUGUGAU | CACAAUCGAAGCAAUGAAAAG |
| circKPNB1-KD2 | UCAAUGUCACUUUUCAUUGCU | CAAUGAAAAGUGACAUUGAUG |
| SPI1-KD1 | UGAGAUAGGGGUAAUACUCGU | GAGUAUUACCCCUAUCUCAGC |
| SPI1-KD2 | UCUUCUUCACCUUCUUGACCU | GUCAAGAAGGUGAAGAAGAAG |
| DGCR8-KD1 | AUUAUUGGAAAUCACAAUGCA | CAUUGUGAUUUCCAAUAAUUG |
| DGCR8-KD2 | UUUUUUCACAGUAACUUGCUC | GCAAGUUACUGUGAAAAAAUU |
| siRNA-NC | UUCUUCGAAGGUGUCACGUTT | ACGUGACACCUUCGAAGAATT |

**Supplementary Table 3. siRNA sequences**
