# Supplementary material for: CircKPNB1 mediates a positive feedback loop and promotes the malignant phenotypes of GSCs via TNF-α/NF-κB signaling
Source: Cell Death Dis. 2022 Aug 9;13(8):697. doi: 10.1038/s41419-022-05149-1 (PMC9363451; doi:10.1038/s41419-022-05149-1)
Supplement: Supplementary file 10 — Table S5 [file 41419_2022_5149_MOESM10_ESM.docx]

**Supplementary Table 5. ChIP qRT-PCR Primers**

| **Primer** | **Forward (5’-3’)** | **Reverse (5’-3’)** |
| --- | --- | --- |
| TNF-α | CGATGAAGCCCAATAAACC | TCTCTGAGCCCTTATCCTT |
| DGCR8 | TTCCACTATTCCTTCCACAT | TCCGAGATAGCACCACTT |
| Nestin | CTGCTGCTTCCACTTCTG | CTGTATAACTGCGAGGTTCA |
